# The potential therapeutic role of itaconate and mesaconate on the detrimental effects of LPS-induced neuroinflammation in the brain

**DOI:** 10.1186/s12974-024-03188-3

**Published:** 2024-08-20

**Authors:** Melanie Ohm, Shirin Hosseini, Niklas Lonnemann, Wei He, Tushar More, Oliver Goldmann, Eva Medina, Karsten Hiller, Martin Korte

**Affiliations:** 1 Department of Cellular Neurobiology, Zoological Institute, TU Braunschweig, 38106 Braunschweig, Germany; 2 Neuroinflammation and Neurodegeneration Group, Helmholtz Centre for Infection Research, 38124 Braunschweig, Germany; 3 Department of Bioinformatics and Biochemistry, Braunschweig Integrated Centre of Systems Biology (BRICS), TU Braunschweig, 38106 Braunschweig, Germany; 4 Infection Immunology Research Group, Helmholtz Centre for Infection Research, 38124 Braunschweig, Germany

**Keywords:** Microglia, LPS, Neuroinflammation, Synaptic plasticity, Hippocampus

## Abstract

Despite advances in antimicrobial and anti-inflammatory treatment, inflammation and its consequences remain a major challenge in the field of medicine. Inflammatory reactions can lead to life-threatening conditions such as septic shock, while chronic inflammation has the potential to worsen the condition of body tissues and ultimately lead to significant impairment of their functionality. Although the central nervous system has long been considered immune privileged to peripheral immune responses, recent research has shown that strong immune responses in the periphery also affect the brain, leading to reactive microglia, which belong to the innate immune system and reside in the brain, and neuroinflammation. The inflammatory response is primarily a protective mechanism to defend against pathogens and tissue damage. However, excessive and chronic inflammation can have negative effects on neuronal structure and function. Neuroinflammation underlies the pathogenesis of many neurological and neurodegenerative diseases and can accelerate their progression. Consequently, targeting inflammatory signaling pathways offers potential therapeutic strategies for various neuropathological conditions, particularly Parkinson’s and Alzheimer’s disease, by curbing inflammation. Here the blood–brain barrier is a major hurdle for potential therapeutic strategies, therefore it would be highly advantageous to foster and utilize brain innate anti-inflammatory mechanisms. The tricarboxylic acid cycle-derived metabolite itaconate is highly upregulated in activated macrophages and has been shown to act as an immunomodulator with anti-inflammatory and antimicrobial functions. Mesaconate, an isomer of itaconate, similarly reduces the inflammatory response in macrophages. Nevertheless, most studies have focused on its esterified forms and its peripheral effects, while its influence on the CNS remained largely unexplored. Therefore, this study investigated the immunomodulatory and therapeutic potential of endogenously synthesized itaconate and its isomer mesaconate in lipopolysaccharide (LPS)-induced neuroinflammatory processes. Our results show that both itaconate and mesaconate reduce LPS-induced neuroinflammation, as evidenced by lower levels of inflammatory mediators, reduced microglial reactivity and a rescue of synaptic plasticity, the cellular correlate of learning and memory processes in the brain. Overall, this study emphasizes that both itaconate and mesaconate have therapeutic potential for neuroinflammatory processes in the brain and are of remarkable importance due to their endogenous origin and production, which usually leads to high tolerance.

**Supplementary Information:**

The online version contains supplementary material available at 10.1186/s12974-024-03188-3.

## Introduction

The central nervous system (CNS), has only a limited capacity for self-regeneration, and must therefore be particularly well protected against potential damage. For a long time, the brain was considered as an immune-privileged organ, due to its separation from the periphery by the blood–brain barrier (BBB) and the absence of lymphatic vessels [1]. However, intensive research demonstrated that the CNS is neither isolated nor passive in its interaction with the immune-system and even in the absence of inflammation the immune system and CNS communicate with each other.

Certain pathogens, including specific viruses and bacterial infections, whether located in the brain or even in periphery, have the potential to impact the CNS. Among those acute systemic inflammatory processes triggered by lipopolysaccharide (LPS), the most common antigen on the cell surface of most Gram-negative bacteria, lead to neuronal death and neurodegeneration, particularly in the hippocampus, a brain area of high importance for processes of learning and memory [2–7]. Immune reactions in the brain are mainly characterized by the reactivity of brain resident immune cells, namely microglia. Microglial reactivity results in production of various cytokines and chemokines as well as the production of complement component 1q (C1q), prostaglandin E2 (PGE2) and free radicals (NO and ROS) [8–10]. In addition to microglia, astrocytes, cells with immunological properties are involved in neuroinflammatory processes. Inflammatory mediators released by microglia can initiate the transformation of astrocytes from a quiescent resting to a reactive phenotype [11], additionally they produce inflammatory mediators actively contributing to the inflammatory status of the brain [12, 13].

Although neuroinflammatory responses are part of the brain’s defense mechanism to eliminate cellular debris and pathogens, as well as facilitate tissue repair, prolonged chronic responses can result in various neuropathological manifestations [14, 15]. For example, neuroinflammation is associated with various neurological disorders such as neurodegenerative diseases like Alzheimer’s disease (AD), Parkinson’s disease (PD), multiple sclerosis and even depression [16–18]. Recent studies suggest that neuroinflammatory processes may not only exacerbate but also trigger the onset of these diseases [15]. Therefore, inflammatory signaling pathways have been implicated as potential therapeutic targets for many neurological diseases to counteract the extent of the inflammation.

Itaconate, a metabolite derived from the tricarboxylic acid cycle (TCA), has attracted considerable interest as an intriguing example of an immunomodulator with anti-inflammatory and antimicrobial properties [19–21]. It is predominantly produced in myeloid cells specifically during inflammation via the inducible aconitate decarboxylase 1 (ACOD1), also known as immune-responsive gene 1 (IRG1) protein [19, 20, 22, 23]. The anti-inflammatory potential of itaconate was first described in *Irg1*-deficient mice (*Irg1*^*−/−*^*)*, unable to convert cis-aconitate to itaconate and therefore have a lack in the itaconate synthesis. Macrophages from these animals showed increased release of inflammatory mediators induced by LPS compared to wild-type (WT) [24]. However, most studies have focused on investigating the effects of chemically esterified forms of itaconate, dimethyl itaconate (DMI) and 4-octyl itaconate (4-OI), which are known for their higher cellular permeability [19]. Recently, it was revealed that DMI and 4-OI possess much higher electrophilicity than the endogenously produced itaconate, and such electrophilicity underlies the ability to activate signaling pathways including nuclear factor erythroid-2-related factor 2 (NRF2) [25]. Therefore, it is necessary to focus on the biological effects of the endogenous form of itaconate rather than the chemical derivatives.

Recently described by He et al., the stimulation of macrophages with LPS results not only in the production of endogenous itaconate but also mesaconate, an isomer of itaconate. The only structural difference between these metabolites is the position of the double bond [19]. Both metabolites have been shown to reduce the inflammatory response in inflammatory peripheral macrophages [19]. In addition, intraperitoneal (i.p) injection of itaconate and mesaconate appears to reduce the extent of LPS-induced systemic inflammation and prolonged survival following a lethal dose of LPS [19]. However, little is known about the effect of these two metabolites within the CNS.

Sun et al. demonstrated that the administration of itaconate has a neuroprotective effect against the extent of PD symptoms in the mouse model [26]. In addition, treatment with itaconate was able to reduce the inflammatory symptoms caused by the reperfusion injury [27]. Therefore, we investigated here whether itaconate without chemical modification can alleviate the inflammatory processes in the rodent brain induced by peripheral immune stimulation and to evaluate its therapeutic role. In addition, the anti-inflammatory properties of mesaconate in various pathological conditions are still not clear.

Since the use of endogenous molecules as therapeutics is of great importance due to their natural production and expected high tolerability, this study investigated the therapeutic potential of itaconate and mesaconate in peripheral LPS-induced neuroinflammation in the particularly susceptible brain region, the hippocampus.

Our results show that pretreatment with exogenous itaconate or mesaconate alleviates LPS-triggered neuroinflammation. This effect is manifested by both the reduced microglial activation and the prevention of LPS-induced synaptic plasticity impairment, highlighting the ability of both metabolites to preserve synaptic functionality.

## Material and methods

### Animals

For the injection of itaconate and mesaconate followed by LPS, 3-month-old C57BL/6J male mice were used. Mice were bred and kept at the animal facility of the TU Braunschweig under standard housing conditions in a 12:12 light:dark cycle at 22 °C with food and water available ad libitum. The performed mice experiments were approved according to the animal welfare law in Germany.

To investigate the impact of *Irg1-gene* on LPS-induced neuroinflammation, *Irg1*^*−/−*^ and WT mice as controls were used in the age of 6 months. Mice were bred and kept at the central mouse facility of the Helmholtz Centre for Infection Research, Braunschweig, Germany.

All protocols used in this project have been reviewed and approved by the local committees at TU Braunschweig and the authorities (LAVES, Oldenburg, Germany 33.19–42502-04–21/3734) according to the national guidelines of the animal welfare law in Germany (‘Tierschutzgesetz in der Fassung der Bekanntmachung vom 18. Mai 2006 (BGBl. I S. 1206, 1313).

### Intraperitoneal injections

Mice were intraperitoneally (i.p.) injected with itaconate and mesaconate on three consecutive days. The dosage of 250 mg/kg bodyweight was used because He et al. demonstrated an immunoregulatory effect at this dosage [19]. Control mice received equal volumes of PBS, the solvent of the metabolites. Prior to the injection mice were checked for possible changes that might affect the experiment. The bodyweight and other external factors were monitored and documented daily. The bodyweight was used to define the appropriate amount of the injection solutions and were injected i.p. accordingly. Subsequently, to induce a systemic immune stimulation 24 h after the last metabolites injection mice received a dual i.p. LPS (E. coli O127:B8, Sigma Aldrich L 3129, 0.5 mg/kg bodyweight) stimulus or saline as control solution, at 24 h intervals [7]. Three hours after the second LPS-injection, mice were sacrificed and samples were collected.

To test the impact of the lack of the *Irg1*-gene in LPS-induced neuroinflammation *Irg1*^−/−^ mice and respective controls were injected two-times with LPS (0.5 mg/kg bodyweight) or an appropriate amount of saline solution as a control in a 24 h interval. Three hours after the second injection mice were sacrificed.

### Primary microglial and astrocytic cultures stimulated with LPS

Primary glial cultures were prepared as described before [7]. Briefly, neonatal mouse brains (P2-P4) of mixed genders, excluding the hippocampus, cerebellum and meninges, were transferred into HBSS 1X on ice. After dissecting the brains mechanically, the tissue was centrifuged 5 min at 300 ×g at 4 °C and the pellet was strained (100 µm pores) after resuspension in HBSS followed by another centrifugation step (400 ×g, 5 min, 4 °C). The resulting pellet was resuspended in 10 mL culture medium (low DMEM + 10% FCS + 1% Penicilin/Streptomycin) and cultured in a poly-lysine coated T-75 flask at 10% CO_2_at 37 °C. The medium was initially replaced after 3 days, followed by a weekly medium change After 2 weeks, once a confluent cell layer formed, microglia were harvested by shaking the flasks at 180 rpm for 3 h at 37 °C and plated in a 12-well plate (5×10^5^ cells/well). Depending on the experiment, following 24 h incubation microglia were either pre-incubated with itaconate or mesaconate (10 mM) for 4 h before LPS (10 ng/mL) was added for further 21 h, or they were only treated with LPS for 24 h, until supernatant was collected and cells were harvested.

Primary astrocytic cultures were obtained from neonatal mouse brains (P1-3) by decapitating, removing the hippocampus, cerebellum and meninges, and processing in fresh HBSS 1X. Following a slide mechanically dissociation, HBSS was replaced by a dissociation solution (9.1 mL DMEM low glucose medium, 400 µL 10 × Trypsin/EDTA, 200 µL 1M HEPES, 5 mg/mL in 0.15 mol/L NaCl DNAse, 100 µl 100 × Penicilin/Streptavidin). The mixture was incubated at 37 °C for 30 min, gently shaken every 4 min, and subsequently further dissociated by pipetting. After a centrifugation step (7 min at 800 rpm), to obtain good dissociation, the glass micro pipettes bore was fire polished to a smaller opening. After discarding the supernatant and adding culture medium (DMEM low glucose, 10% FCS, 1% Penicillin/Streptavidin), the homogenate was passed through a 40 µm cell strainer and the cell suspension was plated in poly-D-lysine-coated T75 flasks. Cultures were incubated at 10% CO_2_, 37 °C for ~ 2 weeks, with a medium change every 2–3 days. Once confluence was reached, overnight shaking at 220 rpm isolated astrocytes. Cells were passaged at a 1:2 ratio after two passages and overnight shaking. Harvested cells were plated in 12-well plates (5×10^5^ cells/well) and, after 24 h, stimulated with 100 ng/mL LPS for 24 h before supernatant collection and cell harvest.

### Stable isotope tracing of LPS-stimulated primary microglia

The tracer medium was prepared using DMEM (GibcoTM) without glucose and glutamine, as well as phenol red. This medium was supplemented with either [U-13C6] glucose (at a final concentration of 5.5 mM) along with unlabeled glutamine (at a final concentration of 4 mM), or [U-13C5] glutamine (at a final concentration of 4 mM) combined with unlabeled glucose (at a final concentration of 5.5 mM). Both tracers were purchased from Cambridge Isotope Laboratories. Initially, cells were cultured in standard low glucose DMEM medium, subsequently replaced with medium equipped with the tracers. The cells were then further incubated for 24 h to achieve isotopic equilibrium before stimulated with LPS.

### Cytokine immunoassay of blood and brain tissue

Cytokine immunoassay of blood and brain tissue was performed using Enzyme-linked immune sorbent assay (ELISA) as described before [28]. The mice were euthanized via CO_2_ asphyxiation, followed by decapitation. The trunk was positioned over a collection tube to allow for blood drainage. The blood was then allowed to clot for 20 min at room temperature (RT), followed by centrifugation at 2000 ×g for 20 min. This protocol ensures the collection of a sufficient volume of blood for the ELISA assay. The whole brain was carefully removed. One hemisphere was frozen in liquid nitrogen and stored at − 80 ℃ until usage. The brain was homogenized by the GentleMACS (Milteny Biotec, Protein_01 program) in 500 µL STKM lysis Buffer (containing: 250 mM sucrose, 50 mM Tris–HCl, 25 mM KCl, and 5 mM MgCl_2_) and protease inhibitor mixture (Roche cOmplete^™^ Protease Inhibitor Cocktail tablet). Subsequently, the samples were centrifuged for 10 min at 13.000 × g at 4 °C. The supernatant was stored at − 70 °C until assayed. Mouse IL-1beta/IL-1F2 DuoSet, Mouse IL-6 DuoSet, Mouse IL-10 DuoSet and mouse TNF-alpha DuoSet ELISA kits (R&D SYSTEMS) were used to determine cytokine levels according to the manufacturer’s recommendations. For measuring cytokines of cell culture supernatants cytokines were diluted 1:5 similar to blood samples. Brain homogenates were diluted 1:2. The absorbance was measured using an Epoch microplate reader at 450 nm connected to the Gen 5 software (BioTek). Lastly, the recorded optical density of the reaction was compared with the optical density of established standard samples in order to ascertain the protein concentration within the test samples. To normalize the detected density of the ELISA the total protein concentrations of the brain tissue samples were measured using a Bradford assay. Briefly, a 1:200 dilution of the samples in 100 µL Bradford solution was added and measured at 595 nm using the Epoch microplate reader.

### Immunohistochemistry

Immunohistochemistry was performed for visualization of different cell types and proteins. Mice were deeply anesthetized by CO_2_ and sacrificed via decapitation. The fresh cerebral hemispheres were fixed in 4% paraformaldehyde (PFA) in PBS for 24 h, followed by cryoprotection in 30% sucrose solution in PBS 1X for at least 24 h. On the day of experiment, the hemispheres were frozen in Tissue-Tek^®^ (Hartenstein Laborversand) at − 70 °C. Frozen brain hemispheres were cut into 30 µm thick slices using a Reichert Jung/Leica Frigocut 2800E cryostat microtome. Five to six sections per mouse were used for free-floating immunohistochemical experiments. The sections were washed twice with PBS 1X for two minutes each and three times with 0.1% Triton X-100 for five minutes. Subsequently, the sections were permeabilized for one hour in blocking solution (0.3% Triton X-100, 10% goat serum, 5% bovine serum albumin (BSA)) followed by incubation of respective primary antibodies (IBA1, 1:1000, rabbit, WAKO 019–19741; CD68 clone FA-11, rat, 1:500; Bio-Rad MCA 1957; GFAP, 1:1000, mouse, Sigma Aldrich G 3893) diluted in blocking solution overnight at 4 °C. The next day sections were washed 3 times with PBS 1X for 10 min each, followed by incubation in respective secondary antibodies diluted 1:500 in PBS 1X for 2 h at RT (Cy^™^3 AffiniPure goat anti-rabbit IgG H + L, 111–165-144; Cy^™^5 AffiniPure goat anti-rat IgG H + L, 112–175-167; Cy^TM^2-conjugated AffiniPure Goat Anti-Mouse IgG H+L, 111–165-144) Finally, the brains slices were washed again three times with PBS 1X for 10 min each, stained with 4′, 6-diamidino-2-phenylindole (DAPI) (Sigma-Aldrich) for 5 min and mounted with Fluoro-gel medium (Electron Microscopy Sciences, Hatfield, PA) on glass slides.

### Golgi-Cox staining

Golgi-Cox staining was performed using the FD Rapid GolgiStain^TM^ Kit (FD Neuro-technologies, Inc.) according to the manufacturer’s protocol. Briefly, mice were deeply anesthetized using CO_2_ and sacrificed via decapitation. The brain was carefully dissected and one of the cerebral hemispheres was incubated in the Golgi solution mixture according to the manufacturer protocol. Before sectioning the brain in 150 µm coronal slices using a Leica Vibratome (VT 1000S), the cerebral hemispheres were embedded in 2% agar. The sections were dried on gelatin-coated slides. In the following steps, the sections were further processed for signal development according to the kit manufacturer’s guidelines. Finally, the sections were mounted using Permount (Thermo Fisher Scientific).

## Electrophysiological recordings

Hippocampi of mice from experimental groups were dissected and cooled in ice-cold carbonated (95% O_2_ and 5% CO_2_) artificial cerebrospinal fluid (ACSF) containing 124 mM NaCl, 4.9 mM KCl, 1.2 mM KH_2_PO_4_, 2 mM MgSO_4_, 2 mM CaCl_2_, 24.6 mM NaHCO_3_ and 10 mM D-glucose. Afterward, 400 µm thick slices were chopped with a manual tissue slicer (Stoelting) and were kept at 32 °C with a constant flow rate (0.5 mL/min) of carbonated ACSF for 2 h until recording. Recordings were performed as previously described [29, 30]. Field excitatory postsynaptic potentials (fEPSPs) were elicited through electrical stimulation along the CA3 to CA1 Schaffer collateral pathway, using a lacquer-coated stainless-steel electrode (5 MΩ; AM Systems). Measurements were taken in the CA1 stratum radiatum, with the recording electrode positioned at least 20 μm from the stratum pyramidale in the apical dendritic layer. The signals captured by the electrode were amplified and digitized with an AM Systems amplifier (model 1700) and a CED 1401 analog-to-digital converter (Cambridge Electronic Design). Basal synaptic transmission was assessed by plotting an input–output curve relating afferent stimulus intensity to the fEPSP slope, with the test stimulus set to evoke fEPSPs at 40% of the maximal response. Short-term plasticity was analyzed by administering two consecutive stimuli of equal intensity across various interpulse intervals (10, 20, 40, 60, 80 and 100 ms). For long-term potentiation (LTP) studies, after establishing a stable baseline, LTP was induced with theta-burst stimulation (TBS) comprising four 100 Hz bursts, repeated 10 times at 200 ms intervals, and applied thrice every 10 s. fEPSP slopes were monitored for an hour post-stimulation and normalized to the pre-TBS baseline. Both acquisition and analysis of the data were carried out using IntraCell software (version 1.5, LIN, Magdeburg, 2000).

### Total RNA and quantitative real time-PCR

RNA extraction was performed by using NucleoSpin^®^ RNA isolation kit (Macherey–Nagel, Düren, Germany). Followed by cDNA synthesis with High Capacity cDNA Reverse Transcription Kit (Thermo Fisher Scientific). Quantitative RT-PCR was performed using TaqMan^®^ using BlueProbe qPCR Mix (Biozym) and using the house keeping control *GAPDH*. Expression levels of target mRNA of *IL-1β, IL-6 and BDNF* was analyzed using the ∆∆Ct method and were normalized to the expression level of the house keeping gene.

Primer sequences bought at EUROFINS:

GAPDH sense (s): GCCTTCCGTGTTCCTACC, antisense (a): CCTCAGTGTAGCCCAAGATG probe (p): CGCCTGGAGAAACCTGCCAAGTA;

IL-1β s: ACGGACCCCAAAAGATGAAG, a: TTCTCCACAGCCACAATGAG, p: AGAGCATCCAGCTTCAAATCTCGCA;

IL-6: s: TGCTACCAAACTGGATATAATCAGG, a: AGGACTCTGGCTTTGTCTTTC, p: CTTCTGGAGTACCATAGCTACCTGGAGT.

### Flow cytometry analysis

Single cell suspension from fresh brain tissue was created using Adult Brain Dissociation Kit (Milteny Biotec, 130–107-677) and the GentleMACS Milteny Biotec), as described before [7, 31] according to the manufacturer´s protocol. Afterwards, the cells were resuspended in FACS staining buffer (PBS 1X + 1% FCS + 0.1% Na-Azide) and stained for 30 min against mouse CD11b-PerCP-Vio700 Clone REA592 (1:50), CD45-APC (1:50), CD68-PE Clone REA835 (1:50) (Milteny Biotec) in a 96-well plate. The flow cytometry was conducted using BD^®^ LSR II Flow Cytometer and analyzed with FlowJo Software (version 10.8.1). Briefly, in the FACS analysis, forward scatter (FSC) and side scatter (SSC) parameters were used to select the region of interest (ROI), focusing on single cells and excluding doublets to capture individual microglia populations. Within these ROIs, cells were analyzed for CD11b and CD45 expression to distinguish microglia (CD11b^+^/CD45_low_) from other monocytes (minimum number of cells: 100.000). Microglia were further analyzed for CD68 expression, a marker for reactive microglia.

### Extraction of intracellular metabolites in microglial cell culture

Metabolic extraction was performed as previously described [19, 32, 33]. Briefly, 5 ×10^5^ microglia on 12 well plates were washed with 0.9% NaCl and quenched with 250 µL -20°C methanol. After adding an equal volume of 4°C deionized water with 1 µg/mL D6-pentane-dioic acid (C/D/N Isotopes) as internal standard plates were maintained on an ice-cold metal plate. Cells were thoroughly scraped and flushed before transferring to tubes containing 250 µL – 20 °C chloroform. Subsequently. The extracts were vortexed at 1400 rpm for 20 min followed by 5 Min centrifugation at 17000 × g, both at 4 °C. 280 µL of the upper aqueous phase (polar phase) was transferred into gas chromatography compatible glass vials containing a micro insert and the samples were lyophilized under vacuum at 4 °C in CentriVap Concentration System (Labconco). The dried samples were capped and stored at 4 °C until measurement. The interphase was collected for RNA isolation.

## Extraction of metabolites from blood and brain tissue

Metabolic extraction from blood serum was performed as previously described [34]. Briefly, 11 µL blood serum combined with 100 µL of an ice-cold extraction solvent (consisting of methanol and water in an 8:1 ratio, maintained at − 20 °C) and 2 µg/mL of D6-glutaric acid as internal standard. The mixture was vortexed (1400 rpm) at 4 °C for 10 min a, then centrifuged (13000 × g, 4 °C) for another 10 min to precipitate proteins and extract metabolites. The supernatants (90 µL) were transferred to glass vials, dried under cold conditions using a speed-vac, and stored at − 20 °C until gas chromatography-mass spectrometry (GC–MS) analysis.

Tissue metabolic extraction was performed as previously described [35]. Briefly, brain tissue samples (30–80 mg) were promptly placed into 2 mL reaction tubes and snap-frozen in liquid nitrogen. The tubes were stored at – 70 °C until further processing. Upon processing, samples were homogenized in a 2 mL tube containing ceramic beads (1.4 mm, QIAGEN) and an extraction fluid containing an internal standard (4 + 1 MeOH:H_2_O and 64 µM 13C-Ribitol) in a volume ratio of 1000 µl per 100 mg of tissue. Subsequently, the brain samples were homogenized in a Retsch MM400 homogenizer. Throughout the remainder of the experiment, the tubes were diligently kept on ice. A second extraction fluid supplemented with a second internal standard (0.1 M HCl containing 2 µg/mL D6 glutaric acid) was added, with the volume ratio being 500 µl per 100 mg of tissue and tubes were vortexed. Finally, chloroform (800 µL per 100 mg of tissue) was added, followed by a 30-s vortexing step and samples were shaken at 1400 rpm at 4 °C for 15 min. After a centrifugation for 5 min at 17000 × g at 4 °C, the supernatants (60 µL) were transferred to glass vials and dried under using a speed-vac (4 °C). The dried samples were stored at − 20°C until GC–MS.

### GC–MS measurement

GC–MS measurement of relative metabolite levels and isotope enrichment was performed as previously described [32]. Dried samples were derivatized with equal amounts of methoxylamine (20 mg/mL in pyridine) and N-methyl-N-(tert-butyldimethylsilyl) trifluoroacetamide (MTBSTFA) or N-Methyl-N-(trimethylsilyl)-trifluoracetamide (MSTFA) using a derivatization robot (Gerstel MPS). A sample volume of 1µL was injected in a SSL injector in splitless mode at 270 °C. GC–MS analysis was performed by an Agilent 7890B gas chromatogram system coupled to an Agilent 5977B GC/MSD (MSD, Agilent Technologies), equipped with a 30 m DB-35MS + 5m Duraguard capillary column (0.25 mm inner diameter, 0.25 µm film thickness). Helium was used as the carrier gas with a constant flow rate of 1 mL/min. Metabolites were detected in either full scan or selected ion mode. Processing of chromatograms and calculation of mass isotopomer distributions and relative quantification of metabolites were performed using the Metabolite Detector software [36].

### Imaging and image analysis

#### Imaging and quantification of microglial cell density

Z-Stack images of immunohistochemically stained sections (five per animal) were acquired with a ZEISS Imaging system (Imager.M2 AXIO) equipped with an ApoTome and a 20 × objective (0.8 NA) at 1µm increments, focusing on the CA1 and DG regions of the hippocampus to assess microglial density and the fluorescence intensity of IBA1, CD68, or GFAP. Imaging settings, including light intensity and exposure time, were uniform across all groups. Slides were coded to conduct the analysis blindly using Fiji software (BioVoxxel). Eight slices were selected and converted to a 2D image by using “Z-Projection” tool with “maximum intensity” setting. Microglial density was analyzed by merging IBA1 and DAPI images, counting all IBA1 + cells with nuclei manually with the “Multi Point” tool, and calculating cell density (cells/mm^2^) in Excel. Fluorescence intensity analysis involved measuring the integrated density of selected areas and subtracting background readings from unstained regions to obtain the fluorescence signal. All data were normalized within each staining to the mean of the control.

### Statistical analysis

Data were presented as mean ± SEM, analyzed and visualized with GraphPad Prism 8 (GraphPad Software, Inc, United States). Differences between groups were assessed using two-way ANOVA (for LPS-injection and metabolite treatment effects), with Fisher's LSD for post-hoc analysis. Exceptions included specific conditions, such as the final 5 min of electrophysiological measurements, where differences between saline and LPS treatments were evaluated using an unpaired t-test. The significance levels are denoted in the figure legends, the significance value described as */#p < 0.05, **/##p < 0.01, ***/###p < 0.001, ****/####p < 0.0001, as well as in extended tables. The “N/n” of the different experimental groups, as well as the statistical test is indicated in the respective Figure legends. All analysis were conducted blindly by the experimenter.

## Results

### Itaconate and mesaconate pretreatment attenuates LPS-induced neuroinflammation

**Fig. 1:**
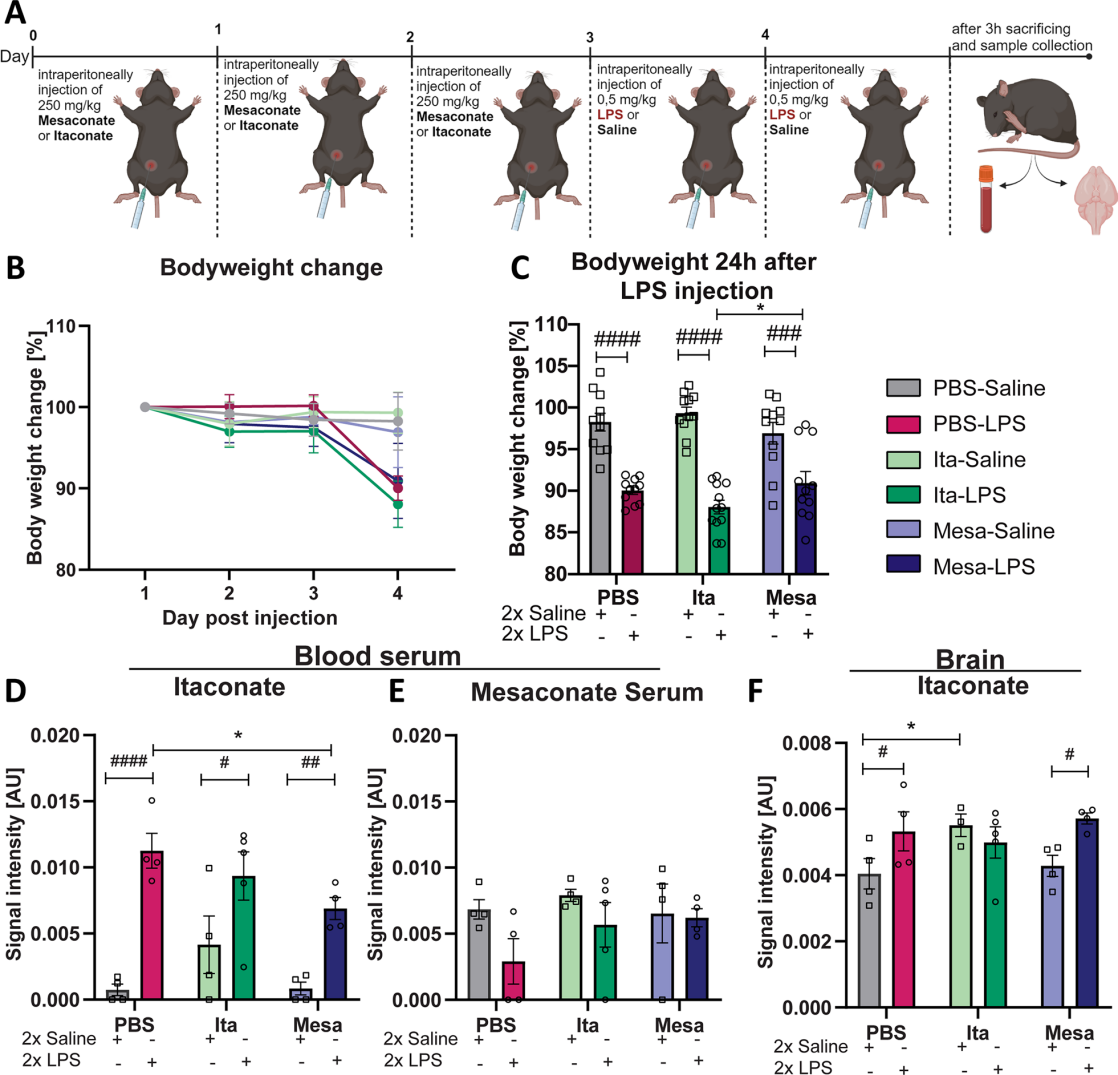
Peripheral LPS injection increases endogenous itaconate production in the blood and brain. **A** Illustration of the injection protocol used in subsequent in vivo studies: Itaconate and mesaconate were administered intraperitoneally (250 mg/kg bodyweight) for three consecutive days, succeeded by two intraperitoneal LPS injections separated by twenty-four hours (0.5 mg/kg bodyweight). Mice were euthanized three hours after the second LPS injection for additional analysis (Created with BioRender.com). **B** Daily body weight monitoring. **C** The bodyweight of the experimental groups was recorded twenty-four hours after the first LPS injection, with the graph showing the percentage change in weight compared to the baseline. **D**, **E** Detected level of itaconate (**D**) and mesaconate (**E**) in the blood across the experimental groups, as well as the detected itaconate level of itaconate in the brain (**F**). Data are presented as mean ± SEM and were analyzed with the with a two-way ANOVA (**C**) followed by Fisher’s LSD test; */#p < 0.05, ##p < 0.01, ###p < 0.001, ####p < 0.0001, **C**: [N (number of mice per group) = 11–12; **D**–**F**: N = 3–5]

To investigate the possible immunomodulatory and preventive effects of itaconate and mesaconate on LPS-induced neuroinflammation, 3-month-old male C57BL/6 mice received three injections of these metabolites (250 mg/kg) or PBS as the solvent of the metabolites as a control solution in a twenty-four-hour interval. Twenty-four hours after the last injection, mice received two injections of either LPS (0.5 mg/kg) or saline, administered twenty-four hours apart. Three hours after the second LPS injection, the mice were sacrificed and the samples were collected (Fig. 1A).

Daily bodyweight monitoring revealed that administration of itaconate and mesaconate did not prevent LPS-induced bodyweight loss, which indicates that the initial immunological response is not compromised (Fig. 1B, C, PBS-Saline vs. PBS-LPS, p < 0.0001; Ita-Saline vs. Ita-LPS p < 0.0001; Mesa-Saline vs. Mesa-LPS p < 0.0001, Table 1). Of note, twenty-four hours after LPS injection, all groups showed significant body weight loss compared to the saline-injected mice. However, the mice pretreated with itaconate showed a significant decrease in bodyweight compared to the mesaconate pretreated mice receiving LPS (Fig. 1C, Ita-LPS vs. Mesa-LPS, p = 0.0474, Table 1).

**Table 1:** Significances Fig. 1

Figure 1C	Bodyweight loss 24h after LPS	F_LPS-injection_ (1, 61) = 104.9, p < 0.0001
		F_treatment_ (2, 61) = 0.1027, p = 0.9025
Figure 1D	Itaconate blood	F_LPS-injection_ (1, 19) = 39.32, p < 0.0001
		F_treatment_ (2, 19) = 2.232, p = 0.1346
Figure 1E	Mesaconate blood	F_LPS-injection_ (1, 19) = 3.3111, p = 0.0846
		F_treatment_ (2, 19) = 0.9566, p = 0.4019
Figure 1F	Itaconate Brain	F_LPS-injection_ (1, 18) = 4.213, p = 0.0550
		F_treatment_ (2, 18) = 0.8434 p = 0.4466

In view of the biologically short half-life of itaconate and mesaconate, the concentrations of these metabolites in the blood serum and brain at the time of sacrificing (3 h after the last LPS or saline injection) were determined using gas chromatography–mass spectrometry (GC–MS) measurements (Fig. 1D–F). In serum, LPS injection resulted in elevated itaconate blood levels across all LPS-injected groups compared to their respective controls, regardless of whether they were pretreated with the metabolites (Fig. 1: D, PBS-Saline vs. PBS-LPS, p < 0.0001; Ita-Saline vs. Ita-LPS p = 0.0145; Mesa-Saline vs. Mesa-LPS p = 0.0079, Table 1). Notably, mesaconate pretreatment led to a reduction in serum itaconate levels in LPS-injected mice compared to those receiving LPS without metabolite pretreatment (Fig. 1D, PBS-LPS vs. Mesa-LPS p = 0.0455, Table 1). Moreover, comparison of saline injected mice with or without metabolite treatment revealed that itaconate pretreated saline controls showed a non-significant trend of higher itaconate levels in the blood compared to the other two saline treated groups (Fig. 1D). However, the mesaconate content in the blood serum was too low to be reliably quantified by GC–MS and no significant differences were found between the groups (Fig. 1E).

In order to investigate whether parts of the injected metabolites penetrate into the brain or if LPS-induced itaconate level in the brain, we conducted metabolite extraction from brain tissue. Notably, mice pretreated with PBS or mesaconate showed a significant increase of itaconate brain levels after LPS injection compared to the respective controls (Fig. 1F, PBS-Saline vs. PBS-LPS, p = 0.0499; Mesa-Saline vs. Mesa-LPS p = 0.0309, Table 1). However, itaconate concentration in the brain of mice pretreated with itaconate showed no significant changes after LPS injection compared to the corresponding mice injected with saline, indicating that LPS injection per se did not increase itaconate concentration in the brain of these mice (Fig. 1F). Remarkably, mice pretreated with itaconate followed by saline injection had higher itaconate concentrations in the brain than mice pretreated with PBS or mesaconate followed by saline injection. This could be the reason why a further significant increase of itaconate in the brain of mice pretreated with itaconate and injected with LPS was not observed (Fig. 1F, PBS-Saline vs. Ita-Saline p = 0.0390, Table 1). Mesaconate was not detectable in the brain. Overall, these results indicate that LPS injection increases itaconate levels in both blood and brain. Intriguingly, mice pretreated with itaconate showed higher levels of itaconate in the brain, suggesting that itaconate may reach the brain and be taken up by resident cells and persist even more than fifty hours after the last injection.

**Fig. 2:**
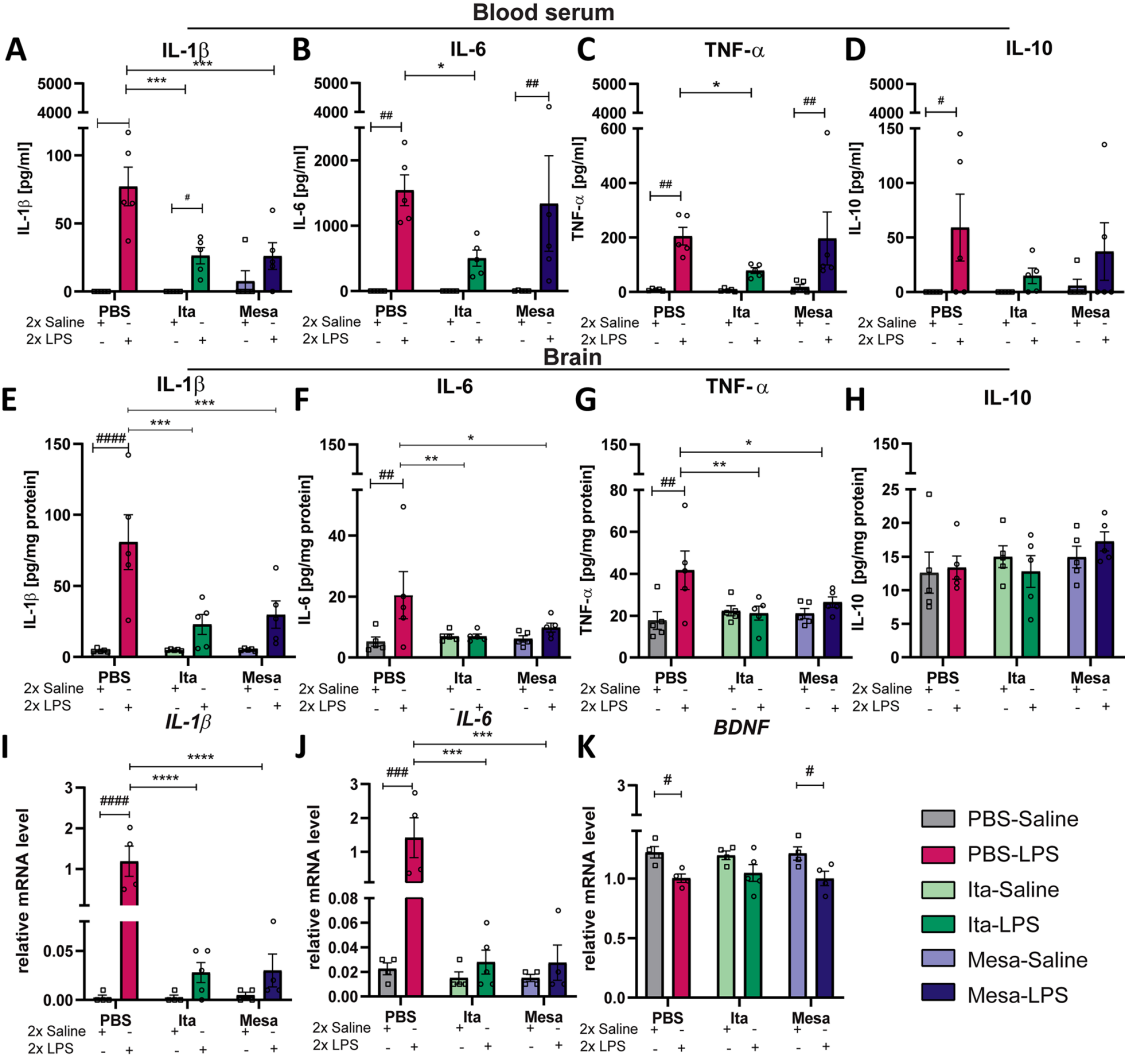
Itaconate and mesaconate ameliorate the release of cytokines in blood and brain after LPS stimulation. Levels of IL-1β, IL-6, TNF-α and IL-10 were assessed in the blood (**A**–**D**) and brain (**E**–**H**) by ELISA. Gene expression levels of IL-1β (**I**), IL-6 (**J**) and BDNF (**K**) were quantified in the hippocampus of all experimental groups using qPCR. Data are presented as mean ± SEM and were analyzed with the two-way ANOVA followed by Fisher’s LSD test; */#p < 0.05, **/##p < 0.01, ***p < 0.001, ####p < 0.0001, [N (number of mice per group) = 4–5]

Bacterial LPS injections induce a robust and transient immune response in both the periphery as well as in the CNS, characterized by an increased release of inflammatory mediators [6, 7, 37]. To evaluate the potential benefits of itaconate and mesaconate in this context, we quantified the levels of secreted specific cytokines in blood serum and brain homogenates using enzyme-linked immunosorbent assay (ELISA) (Fig. 2).

In the absence of itaconate and mesaconate, LPS injection significantly increased the levels of all tested inflammatory mediators in blood serum compared to saline-injected controls (Fig. 2, PBS-Saline vs. PBS-LPS, A: IL-1β p < 0.0001, B: IL-6 p = 0.0022, C: TNF-α p = 0.0031, D: IL-10 p = 0.0211, Table 2). Conversely, in mesaconate pretreated mice, only IL-6 and TNF-α levels increased significantly post-LPS, whereas mice pretreated with itaconate showed only a significant increase in IL-1β upon LPS-challenge (Fig. 2, Mesa-Saline vs. Mesa-LPS, B: IL-6 p = 0.0066, C: TNF-α p = 0.0066; Ita-Saline vs. Ita-LPS, A: IL-1β p = 0.0305, Table 2). Remarkably, itaconate pretreatment significantly reduced LPS-induced release of IL-6 and TNF-α compared to mice injected with LPS without metabolite pretreatment (Fig. 2, PBS-LPS vs. Ita-LPS, A: IL-1β p = 0.0002, B: IL-6 p = 0.0297, C: TNF-α p = 0.0460, PBS-LPS vs. Mesa-LPS, A: IL-1β p = 0.0007, Table 2).

**Table 2:** Significances Fig. 2

Figure 2A	IL-1β—Serum	F_LPS-injection_ (1, 24) = 37.85, p < 0.0001
		F_treatment_ (2, 24) = 5.774, p = 0.0090
Figure 2B	IL-6—Serum	F_LPS-injection_ (1, 24) = 18.90, p = 0.0002
		F_treatment_ (2, 24) = 1.503, p = 0.2427
Figure 2C	TNF-α—Serum	F_LPS-injection_ (1, 24) = 18.61, p = 0.0002
		F_treatment_ (2, 24) = 1.528, p = 0.2374
Figure 2D	IL-10—Serum	F_LPS-injection_ (1, 24) = 6.44, p = 0.0181;
		F_treatment_ (2, 24) = 0.8772
Figure 2E	IL-1β—Brain	F_LPS-injection_ (1, 24) = 27.62, p < 0.0001
		F_treatment_ (2, 24) = 5.749 p = 0.0091
Figure 2F	IL-6—Brain	F_LPS-injection_(1, 24) = 5.371, p = 0.0293
		F_treatment_ (2, 24) = 1.781 p = 0.1900
Figure 2G	TNF-α—Brain	F_LPS-injection_ (1, 24) = 6.100
		F_treatment_ (2, 24) = 1.560 p = 0.2306
Figure 2H	IL-10—Brain	F_LPS-injection_ (1, 24) = 0.03026, p = 0.8634
		F_treatment_ (2, 24) = 1.219 p = 0.3131
Figure 2I	IL-1β—Hippocampus	F_LPS-injection_ (1, 19) = 10.33, p = 0.0026
		F_treatment_ (2, 19) = 10.33, p = 0.0009
Figure 2J	IL-6—Hippocampus	F_LPS-injection_ (1, 19) = 6.342, p = 0.0209
		F_treatment_ (2, 19) = 6.037, p = 0.0093
Figure 2K	BDNF—Hippocampus	F_LPS-injection_ (1, 19) = 18.24, p = 0.0004
		F_treatment_ (2, 19) = 0.04105, p = 0.9599

To assess the neuroinflammatory response to peripheral LPS exposure, inflammatory mediators in the whole brain homogenates were measured (Fig. 2E–H). LPS-injection in PBS-pretreated mice significantly increased levels of IL-1β, IL-6 and TNF-α in the brain compared to saline controls (Fig. 2; PBS-Saline vs. PBS-LPS: E: IL-1β p < 0.0001, F: IL-6 p = 0.0035, G: TNF-α p = 0.0013, Table 2). We did not observe an increase in inflammatory mediators in mice pretreated with itaconate and mesaconate compared to the respective saline controls. Metabolite pretreatment resulted in significantly lower levels of IL-1β, IL-6 and TNF-α in brain compared to PBS-pretreated LPS-challenged mice (Fig. 2, PBS-LPS vs. Ita-LPS: E: IL-1β p = 0.0002, F: IL-6 p = 0.0086, G: TNF-α p = 0.0049; PBS-LPS vs. Mesa-LPS: E: IL-1β p = 0.0007, F: IL-6 p = 0.0329, G: TNF-α p = 0.0304, Table 2). These results were further supported by gene expression analyses for IL-1β and IL-6 in the hippocampus, demonstrating a significant reduction in itaconate- and mesaconate pretreated mice compared to LPS injected mice without metabolite administration (Fig. 2, PBS-LPS vs. Ita-LPS, I: IL-1β p < 0.0001, J, IL-6 p = 0.0003; PBS-LPS vs. Mesa-LPS, I: IL-1β p < 0.0001, J: IL-6 p = 0.0005, Table 2). These findings suggest that itaconate and mesaconate pretreatment can attenuate LPS-induced inflammatory responses, particularly in the brain, highlighting their potential to ameliorate neuroinflammatory processes. Regarding the anti-inflammatory cytokine IL-10, LPS only caused a significant increase in the blood serum in the absence of itaconate and mesaconate, which we did not observe in the other test groups. The IL-10 level in the brain showed no significant differences between the groups, which may indicate that both metabolites alleviate inflammation via modulation of pro-inflammatory mediators rather than anti-inflammatory mediators.

Further, as the brain-derived neurotrophic factor (BDNF) can also attenuate neuroinflammation [38], the possibility of itaconate and mesaconate to regulate BDNF levels in the brain was evaluated. For this purpose, we analyzed BDNF gene expression levels in the hippocampus after LPS injection in all experimental groups. LPS injection significantly lowered BDNF levels in mice pretreated with either PBS or mesaconate, in contrast to saline-injected controls (Fig. 2K, PBS-Saline vs. PBS-LPS, p = 0.0129; Mesa-Saline vs. Mesa-LPS, p = 0.0158, Table 2). However, in itaconate-pretreated mice, the reduction in BDNF expression following LPS injection was not statistically significant compared to saline controls (Fig. 2K, Ita-Saline vs. Ita-LPS, p = 0.0622, Table 2), indicating no significant difference when comparing LPS-injected mice pretreated with itaconate to those treated with saline. These findings suggest that while itaconate and mesaconate mitigated the LPS-induced upregulation of inflammatory mediators indicating their protective potential, itaconate only shows a trend towards a protective effect against LPS-induced reduction in BDNF expression, which may be of interest to investigate in detail in future studies.

### Itaconate and mesaconate pretreatment attenuates LPS-induced microglial reactivity

**Fig. 3:**
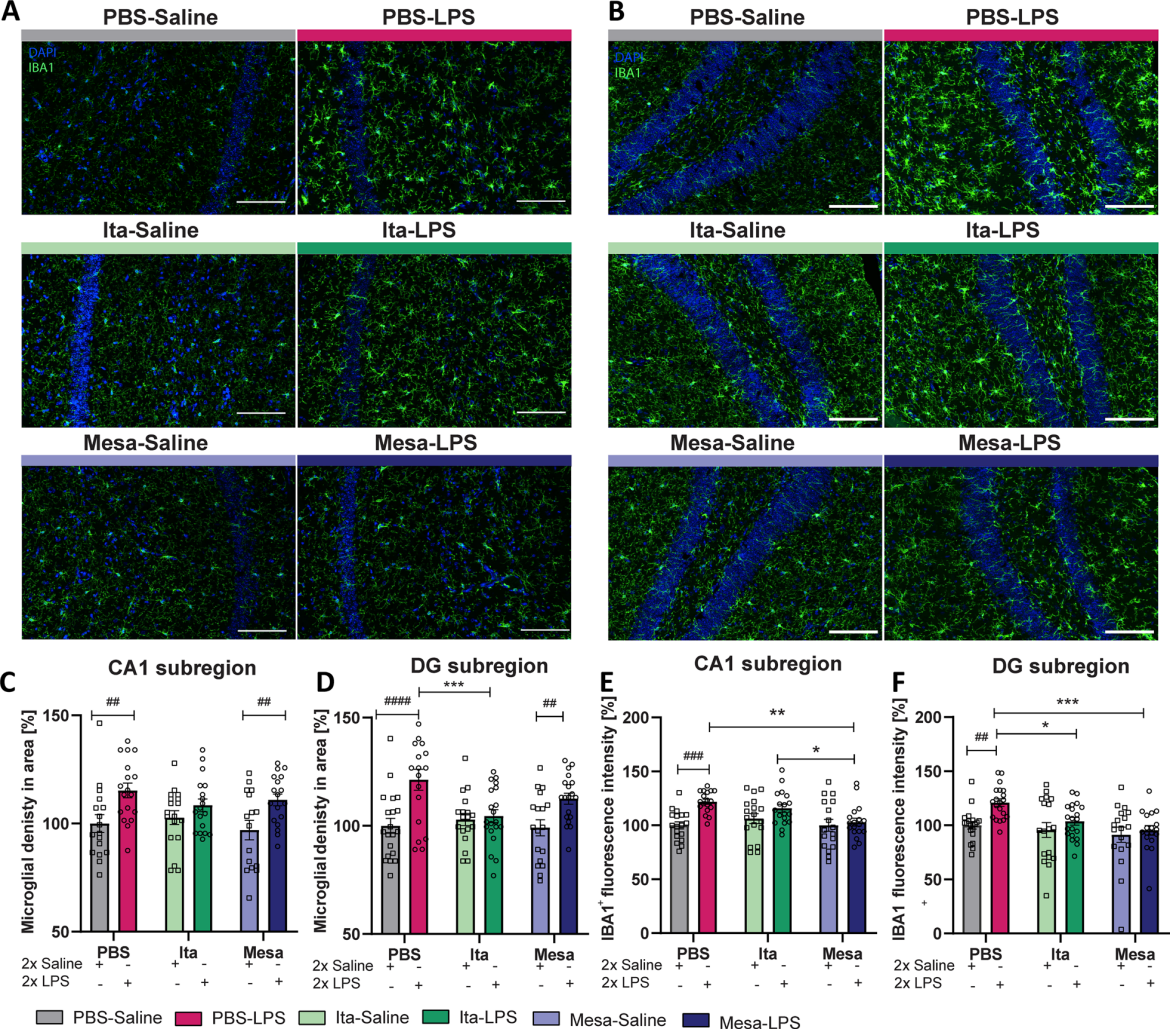
Itaconate and mesaconate dampen the local hippocampal inflammatory effects in microglia after LPS stimulation. Representative images showing immunohistochemical staining for IBA1 and DAPI in the CA1 (**A**) and dentate gyrus (DG) (**B**) region of the hippocampus for all experimental groups (magnification 20x, scale bar is 100 µm). The density of microglia was evaluated in the CA1 (**C**) and DG (**D**) of the hippocampus. Measurement of IBA1 fluorescence intensity in the CA1 (**E**) and DG (**F**). Data are presented as mean ± SEM and were analyzed with the two-way ANOVA followed by Fisher’s LSD test; *p < 0.05, **/##p < 0.01, ***/###p < 0.001, ####p < 0.0001, [N (number of mice per group) = 4 per group, Microglial density CA1 n (number of analyzed imaged per group) = 16–18; DG n = 18–20; IBA1^+^ fluorescence CA1 n = 17–18; DG n = 18–20]. N = 4 per group, CA1, n (number of analyzed imaged per group) = 17–18; DG, n = 17–20; E: N = 6–7,]

Microglia, the brain's resident immune cells, exhibit high sensitivity to environmental alterations, with an elevation in pro-inflammatory mediators prompting a transition into a reactive phenotype [39, 40]. Having shown that the pretreatment with itaconate and mesaconate reduced the extent of inflammatory mediators in both periphery as well as in the brain following LPS exposure, in the following step we investigated whether the metabolite pretreatment is also sufficient in mitigating LPS-induced microglial reactivity. Elevated microglial density and increased IBA1 expression are recognized biomarkers for neuroinflammation and increased microglial reactivity [41, 42]. Therefore, microglial density was quantified by labeling microglia against ionized calcium-binding adapter protein (IBA1), a cytoplasmic marker expressed in both microglia and macrophages, across the CA1 and dentate gyrus (DG) subregions of the hippocampus (representative images for CA1 and DG are shown in Fig. 3A, B, respectively). LPS injection increased microglia density in the CA1 and DG of the hippocampus of PBS-pretreated mice compared to saline-injected controls, which was also present in LPS-injected mice pretreated with mesaconate (Fig. 3C, D; PBS-Saline vs. PBS-LPS: CA1 p = 0.0024, DG p < 0.0001; Mesa-Saline vs. Mesa-LPS: CA1 p = 0.0064, DG p = 0.0076, Table 3).

**Table 3:** Significances Figs. 3 and 4

Figure 3C	IBA1 cell density CA1	F_LPS-injection_ (1, 99) = 16.77, p < 0.0001
		F_treatment_ (2, 99) = 0.5576, p = 0.5744
Figure 3D	IBA1 cell density DG	F_LPS-injection_(1, 107) = 18.32, p < 0.0001
		F_treatment_ (2, 107) = 2.158, p = 0.1205
Figure 3E	IBA1 fluorescence intensity CA1	F_LPS-injection_(1, 101) = 12.81, p < 0.0005
		F_treatment_(2, 101) = 3.873, p = 0.0240
Figure 3F	IBA1 fluorescence intensity DG	F_LPS-injection_(1, 108) = 7.583, p < 0.0069
		F_treatment_(2, 108) = 6.300., p = 0.0026
Figure 4C	CD68 fluorescence intensity CA1	F_LPS-injection_(1, 101) = 1.042, p = 0.3098
		F_treatment_(2, 101) = 1.643, p = 0.1986
Figure 4D	CD68 fluorescence intensity DG	F_LPS-injection_(1, 105) = 4.208, p = 0.0427
		F_treatment_(2, 105) = 3.293., p = 0.0410
Figure 4F	FACS CD68	F_LPS-injection_(1, 35) = 7.988, p = 0.0077
		F_treatment_ (2, 35) = 0.7647, p = 0.4731

However, pretreatment with itaconate prevented this LPS-induced increase in microglia density. Remarkably, a comparison of the LPS-injected groups with or without metabolite pretreatment showed a significant reduced number of IBA1^+^ cells in the DG subregion upon itaconate pretreatment, while mesaconate pretreatment displayed only a slight decrease in LPS-induced microglia density (Fig. 3D, PBS-LPS vs. Ita-LPS, p = 0.0008, Table 3).

In a next step we aimed to evaluate whether itaconate and mesaconate pretreatment mitigates LPS-induced upregulation of IBA1, potentially indicating diminished microglial reactivity (Fig. 3E, F). PBS-pretreated mice injected with LPS showed significant increase in IBA1-fluorescent intensity relative to their saline controls (Fig. 3, PBS-Saline vs. PBS-LPS, E: CA1 p = 0.0001, F: DG: p = 0.0025, Table 3). Interestingly, in mice pretreated with itaconate or mesaconate, we could not observe a significant elevation in IBA1 expression compared to their respective controls. Pretreatment with mesaconate significantly attenuated the LPS-induced increase in IBA1 fluorescence intensity across both hippocampal regions, while itaconate specifically reduced it in the DG compared to PBS-pretreated mice (Fig. 3E, F, PBS-LPS vs. Ita-LPS, DG p = 0.012, PBS-LPS vs. Mesa-LPS, CA1 p = 0.009, DG p = 0.0004, Table 3). Collectively, these results suggest the efficacy of itaconate and mesaconate in preventing the LPS-induced increase microglial reactivity, with itaconate additionally showing the capability to reduce microglial density.

**Fig. 4:**
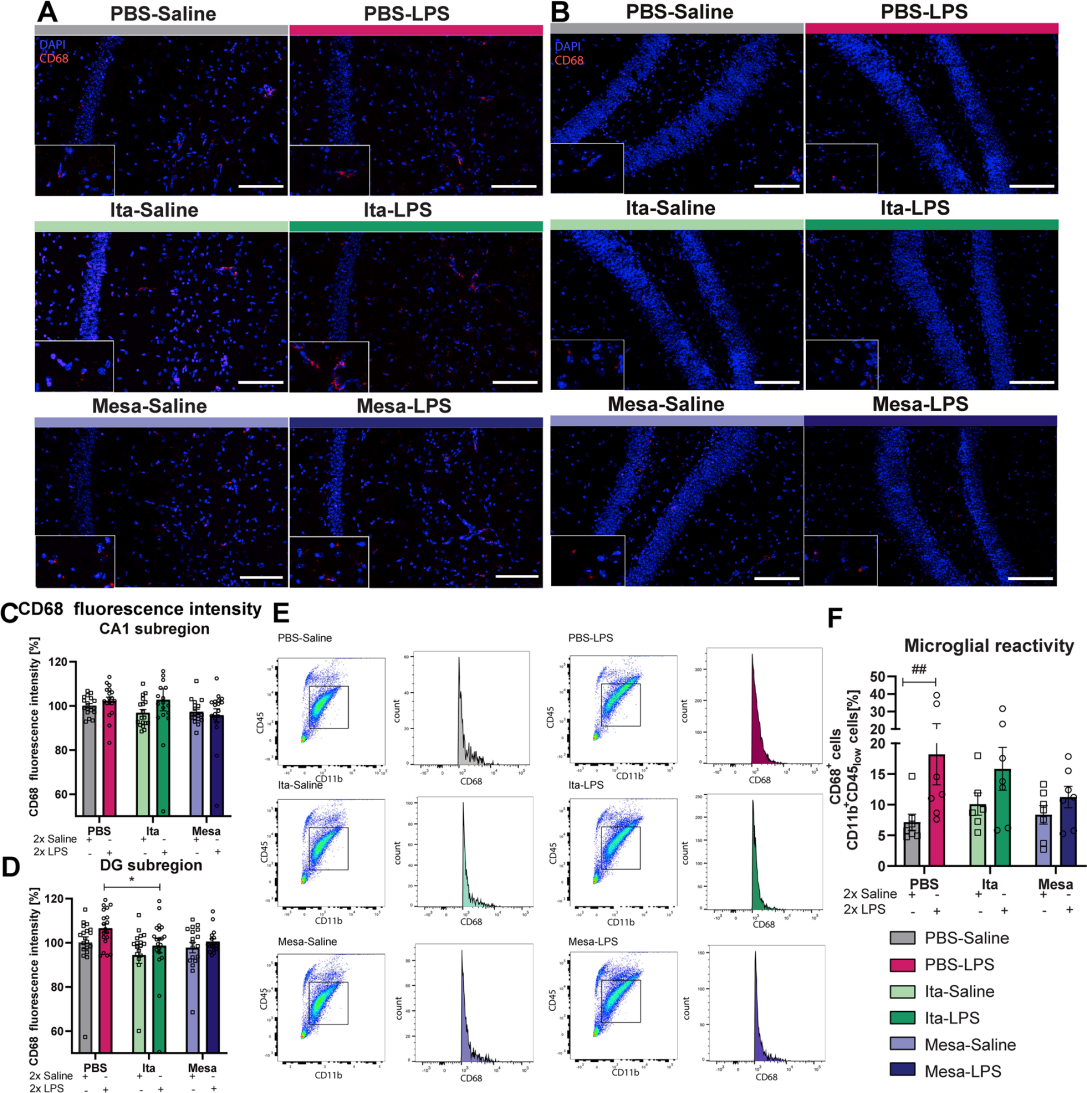
Itaconate and mesaconate slightly dampen the local hippocampal inflammatory effects in microglia after LPS stimulation. Representative images showing immunohistochemical staining for CD68 and DAPI in the CA1 (**A**) and dentate gyrus (DG) (**B**) region of the hippocampus for all experimental groups (magnification 20x, scale bar is 100µm). CD68 fluorescence intensity in the hippocampal CA1 (**C**) and DG (**D**) regions for all experimental groups. Plots representing gated cell population identified as microglia and histogram illustration of CD68 expression determined by FACS. Microglia were characterized by intermediate expressions of CD45^low^ and CD11b^+^. Cells showing CD68 expression above a threshold were classified as CD68^+^-expressing microglia (**E**). Analysis of CD68 expressing microglia cells (**F**). Data are presented as mean ± SEM and were analyzed with the two-way ANOVA followed by Fisher’s LSD test; *p < 0.05, ##p < 0.01, [**C**, **D**: N (number of mice per group) = 4 per group, CA1, n(number of analyzed imaged per group) = 17–18; DG, n = 17–20; F: N = 6–7]

To elucidate the impact of itaconate and mesaconate on microglial reactivity, we examined their effects on the activation marker Cluster of Differentiation 68 (CD68). CD68, a lysosomal protein upregulated in mononuclear cells during inflammation, is a useful microglial activation marker [43]. First, immunohistochemical staining for CD68 was performed (representative images for CA1 and DG are shown in Fig. 4A, B, respectively). CD68 fluorescence intensity analysis indicated that LPS injection did not significantly affect the CA1 subregion, irrespective of itaconate or mesaconate pretreatment (Fig. 4C). However, itaconate pretreatment injected with LPS significantly lowered CD68 intensity in the DG subregion compared to LPS-injected mice pretreated with PBS (Fig. 4D, PBS-LPS vs. Ita-LPS, p = 0.0358, Table 3), while mesaconate showed only a slight decrease. Further, fluorescence-activated cell sorting (FACS) analysis quantified CD68 in whole brain hemisphere-derived single cell suspensions, stained for CD11b, CD45 and CD68. Due to the relatively small sample mass when only the hippocampi are used and a relatively small effect visible in the hippocampus by immunohistochemistry, FACS analysis was performed on whole brain homogenates. Microglia were identified by intermediate CD45 and positive CD11b expression, classified into CD68^+^ or CD68^−^ cells (Fig. 4E). LPS injection increased the number of CD68^+^ microglia compared to saline controls, but not in mice pretreated with itaconate or mesaconate (Fig. 4F). Mesaconate pretreatment even slightly reduced the percentage of CD68^+^ microglia compared to PBS pretreatment, though without statistical significance (Fig. 4F).

While microglia are recognized as the brain's primary immune cells, research has also highlighted astrocytes' significant role in immune responses and regulation, and both glial cells seem to influence each other [11, 44]. In response to neuroinflammation, astrocytes undergo astrogliosis, marked by an increase in glial fibrillary acidic protein (GFAP) expression [45]. To investigate whether pretreatment with itaconate and mesaconate can mitigate LPS-triggered astrogliosis, we conducted immunohistochemical staining for GFAP—a protein found in the cytoplasm of astrocytes—within the hippocampus (representative images for CA1 and DG are shown in Suppl 1A and Suppl. 1B, respectively). LPS-injection resulted only in a slight statistically insignificant increase in GFAP^+^ fluorescence intensity in the DG (Suppl. 1D) which was even less evident in the CA1 region (Suppl. 1C) of PBS-pretreated mice compared to saline-injected controls. This was also the case for itaconate pretreated mice which showed a slight, non-significant elevation in the GFAP fluorescence intensity in comparison to saline injected controls. However, LPS injection did not lead to a similar slight increase of GFAP in mesaconate pretreated mice which was even slightly decreased in the CA1 region (Suppl. 1C). Furthermore, comparing all LPS-injected groups, with or without metabolite pretreatment, revealed that prior exposure to mesaconate resulted in a significant reduction in GFAP fluorescence intensity in the DG compared to LPS-injected mice pretreated with PBS (Suppl. 1D, PBS-LPS vs. Mesa-LPS, p = 0.0495, Table 4). These results indicate that pretreatment with mesaconate was even able to reduce the slight increase in GFAP fluorescence intensity induced by LPS injection in the DG.

**Table 4:** Significances Suppl. Figure 1

Suppl. 1C	GFAP fluorescence intensity CA1	F_LPS-injection_(1, 114) = 0.01229, p = 0.9119
		F_treatment_(1, 114) = 0.3035, p = 0.7388
Suppl. 1D	GFAP fluorescence intensity DG	F_LPS-injection_(1, 106) = 2.649, p = 0.1066
		F_treatment_(2, 106) = 2.683, p = 0.0730

Overall, these results suggest that pretreatment of mice with itaconate or mesaconate is able to attenuate the increased microglial reactivity induced by LPS, thus supporting their anti-inflammatory properties. This is associated with a less pronounced effect on astrocytic reactivity.

### Itaconate and mesaconate pretreatment rescued the LPS-induced impairment of synaptic plasticity

**Fig. 5:**
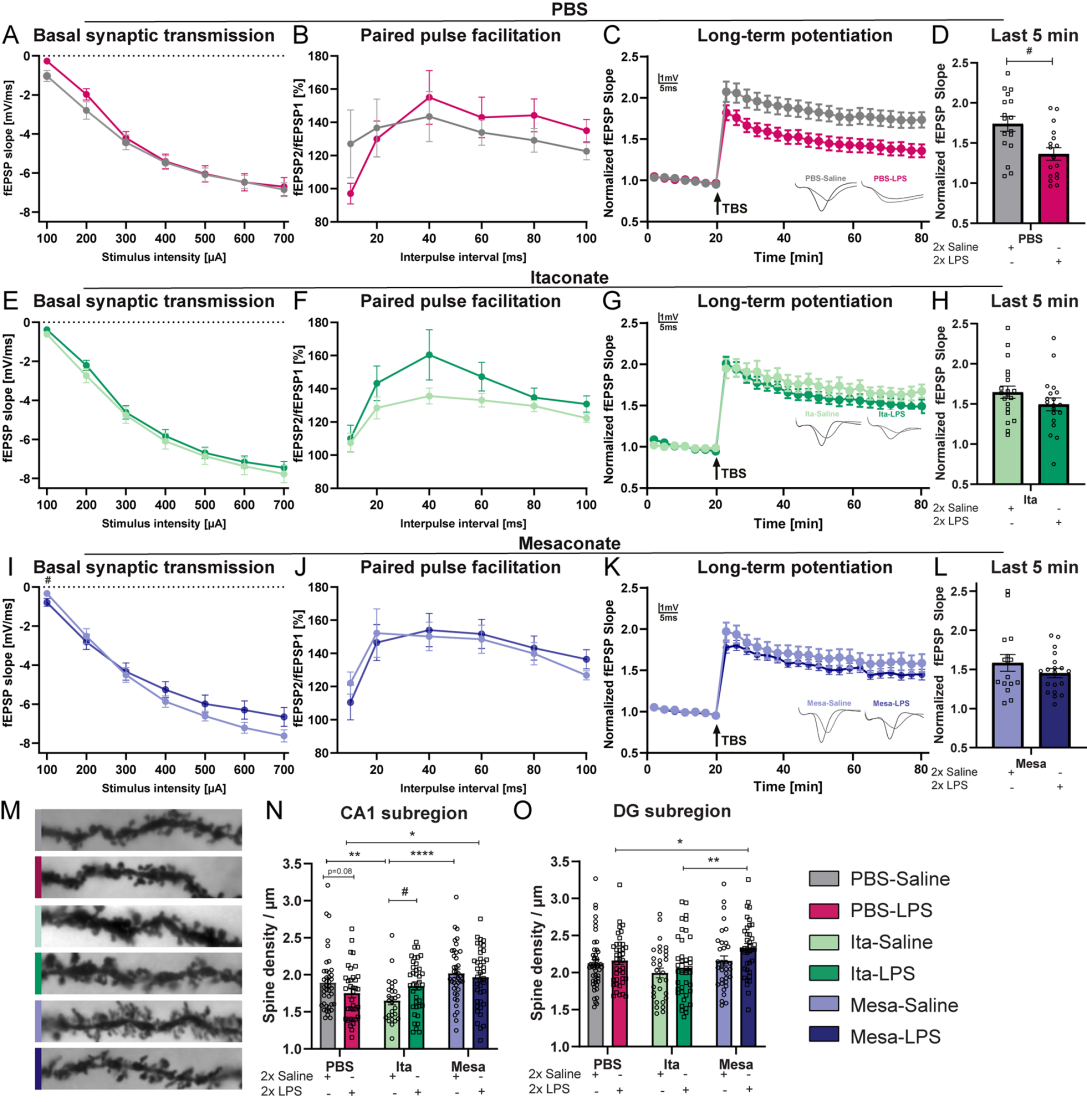
Pretreatment with itaconate and mesaconate prevents LPS-induced impairment of LTP. **A**, **E**, **I** Input–output curves of fEPSP slopes in acute hippocampal slices. **B**, **F**, **J**. Paired-pulse facilitation (PPF) of the fEPSP slopes plotted as a response to the second stimulation over the first one at different interpulse intervals (10, 20, 40, 60, 80, and 100 ms. **C**, **G**, **K** LTP was measured in hippocampal acute slices of all experimental groups. **D**, **H**, **L** LTP maintenance phase (T 75–80 min). **M** Representative images of the Golgi-Cox staining in hippocampal apical dendrites of CA1 subregion of all experimental groups (scale bar is 5 µm). Hippocampal dendritic spine density in CA1 (**N**) and DG (**O**) across the experimental groups. Data are presented as mean ± SEM and were analyzed with a repeated measure two-way ANOVA followed by Fisher’s LSD test (**A, B, C, E, F, G, I, J, K**) or two-way ANOVA followed by Fisher’s LSD test (**N**, **O**) or unpaired t-test (**D**, **H**, **L**); */#p < 0.05, **p < 0.01, ****p < 0.0001, [**A**–**L**: N (number of mice per group) = 4–5, n (number of analyzed hippocampal slices per group) = 16–20; N + O: N = 3–4, n = 27–41]

Previously it was shown that long-term potentiation (LTP), a key form of activity-dependent synaptic plasticity is impaired following a peripheral immune stimulation by LPS-injection in mice [6, 7, 46]. Therefore, after demonstrating an anti-inflammatory potential of itaconate and mesaconate in the brain, we aimed to determine whether pretreatment with itaconate and mesaconate could mitigate the LPS-induced impairment on hippocampal network function. Therefore, electrophysiological recordings were performed as previously described [7, 29, 30] at the Schaffer collateral pathway connecting the CA3 and CA1. Initially, we analyzed the relationship between the fEPSP slope and stimulation intensity using input–output curves. (Fig. 5A, E, I, Table 5). The LPS injection had no significant effect on the basal synaptic transmission compared to saline injected mice, irrespective of prior metabolite treatment. Nonetheless, at a stimulus intensity of 100 μA, mice pretreated with mesaconate and subsequently injected with LPS exhibited a marginally decreased fEPSP slope relative to control mice (Fig. 5I, Mesa-Saline vs. Mesa-LPS, 100 μA stimulus, p = 0.0452, Table 5).

**Table 5:** Significances Fig. 5

Figure 5A	Basal synaptic transmission PBS-Groups	F_LPS-injection_(1, 32) = 0.4627, p = 0.5013
		F_stimulus_(1.592, 50.93) = 224.5, p < 0.0001
Figure 5B	Paired Pulse Facilitation PBS-Groups	F_LPS-injection_(1, 18) = 0.01634, p = 0.8997
		F_stimulus_(2.413, 43.43) = 6.576, p = 0.0019
Figure 5E	Basal synaptic transmission Ita-Groups	F_LPS-injection_(1, 37) = 0.4155, p = 0.5232
		F_treatment_(2,036, 75,32) = 348,4, p < 0,0001
Figure 5F	Paired Pulse Facilitation Ita-Groups	F_LPS-injection_(1, 30) = 1.712, p = 0.2007
		F_treatment_F (1,718, 51,53) = 18,78, p < 0.0001
Figure 5I	Basal synaptic transmission Mesa-Groups	F_LPS-injection_(1,33) = 0.6316, p = 0.4324
		F_treatment_(1,973, 65,12) = 262,8, p < 0.0001
Figure 5J	Paired Pulse Facilitation Mesa-Groups	F_LPS-injection_(1,29) = 0.008853, p = 0.9257
		F_treatment_(2,167, 62,84) = 14,15, p < 0.0001
Figure 5N	Dendritic spine density CA1	F_LPS-injection_(1, 215) = 0.0006828, p = 0.9792
		F_treatment_ (2, 215) = 8.416, p = 0.0003
Figure 5O	Dendritic spine density DG	F_LPS-injection_(1, 223) = 3.514, p = 0.0622
		F_treatment_ (1, 223) = 5.588, p = 0.0043

Subsequent, the potential effects of LPS-injection on short-term synaptic plasticity of CA1 neurons measured by paired pulse facilitation (PPF) was investigated. No significant differences in PPF were detected between the LPS- and saline-injected mice, regardless if they received a metabolite pretreatment or not (Fig. 5B, F, J). These findings suggest that LPS injection does not significantly affect basal synaptic transmission or presynaptic function, and that pretreatment with itaconate or mesaconate does not elicit substantial modifications in these parameters.

Furthermore, long-term synaptic plasticity was examined in the Schaffer collateral CA3 to CA1 pathway induced by TBS, following 20 min of baseline recordings (Fig. 5C, G, K). Consistent with previous studies [6, 7, 46], LTP induction was significantly impaired in the hippocampus of LPS-injected mice compared to saline-injected controls, when pretreated with PBS (Fig. 5C), a phenomenon extending into the LTP maintenance phase (Fig. 5D, p = 0.0101, Table 5). Remarkably, the LPS-induced LTP deficit was not evident in acute hippocampal slices from mice pretreated with either itaconate or mesaconate (Fig. 4G, K), Also, no significant impairment of LTP in the maintenance phase was observed in mice pretreated with metabolites (Fig. 5H, L). Collectively, these findings indicate that pretreatment with itaconate and mesaconate effectively prevent the LPS-induced impairments in LTP, highlighting their potential in preserving the cellular foundations of learning and memory.

In addition to assess neuronal functions, we next evaluated the effect of itaconate and mesaconate on the LPS-induced changes in the neuronal structure. For this purpose, we analyzed the hippocampal dendritic spine density by Golgi-Cox staining (representative images shown in Fig. 5M). The results demonstrated that a dual LPS-stimulus did not lead to a significant decrease in dendritic spine density neither in CA1 (Fig. 5N) nor in DG (Fig. 5O). However, neurons of LPS-injected mice pretreated with PBS revealed a slightly lower dendritic spine density in CA1 neurons compared to its respective control. Conversely, itaconate pretreated mice injected with LPS showed a significant higher dendritic spine density in CA1 neurons than their saline-injected mice (Fig. 5N, Ita-Saline vs. Ita-PBS, p = 0.0310, Table 5). Additionally, in mice pretreated with mesaconate, LPS injections did not lead to a decrease in dendritic spine density in CA1 neurons and actually resulted in an increase in spine density in DG neurons, when compared to control mice injected with saline (Fig. 5O, Saline-Mesa vs. LPS-Mesa p = 0.0508, Table 5). Comparing mice injected with saline, with or without itaconate or mesaconate pretreatment, the results demonstrated that pretreatment with itaconate alone led to a decrease in dendritic spine density in CA1 neurons, in contrast to both PBS-pretreated and mesaconate-pretreated mice (Fig. 5N, PBS-Saline vs Ita-Saline, p = 0.0076; Ita-Saline vs. Mesa-Saline, p < 0.0001, Table 5). Additionally, mice treated with Mesa-LPS showed an increased dendritic spine density in the DG region compared to those treated with Ita-LPS (Fig. 5O, LPS-Ita vs. LPS-Mesa, p = 0.0023, Table 5). These findings indicate that although LPS-induce impairments in LTP, the dual LPS-stimulus do not induce a strong dendritic spine loss.

### Itaconate and mesaconate reduce the LPS-induced inflammation in microglia but not in astrocytes

**Fig. 6:**
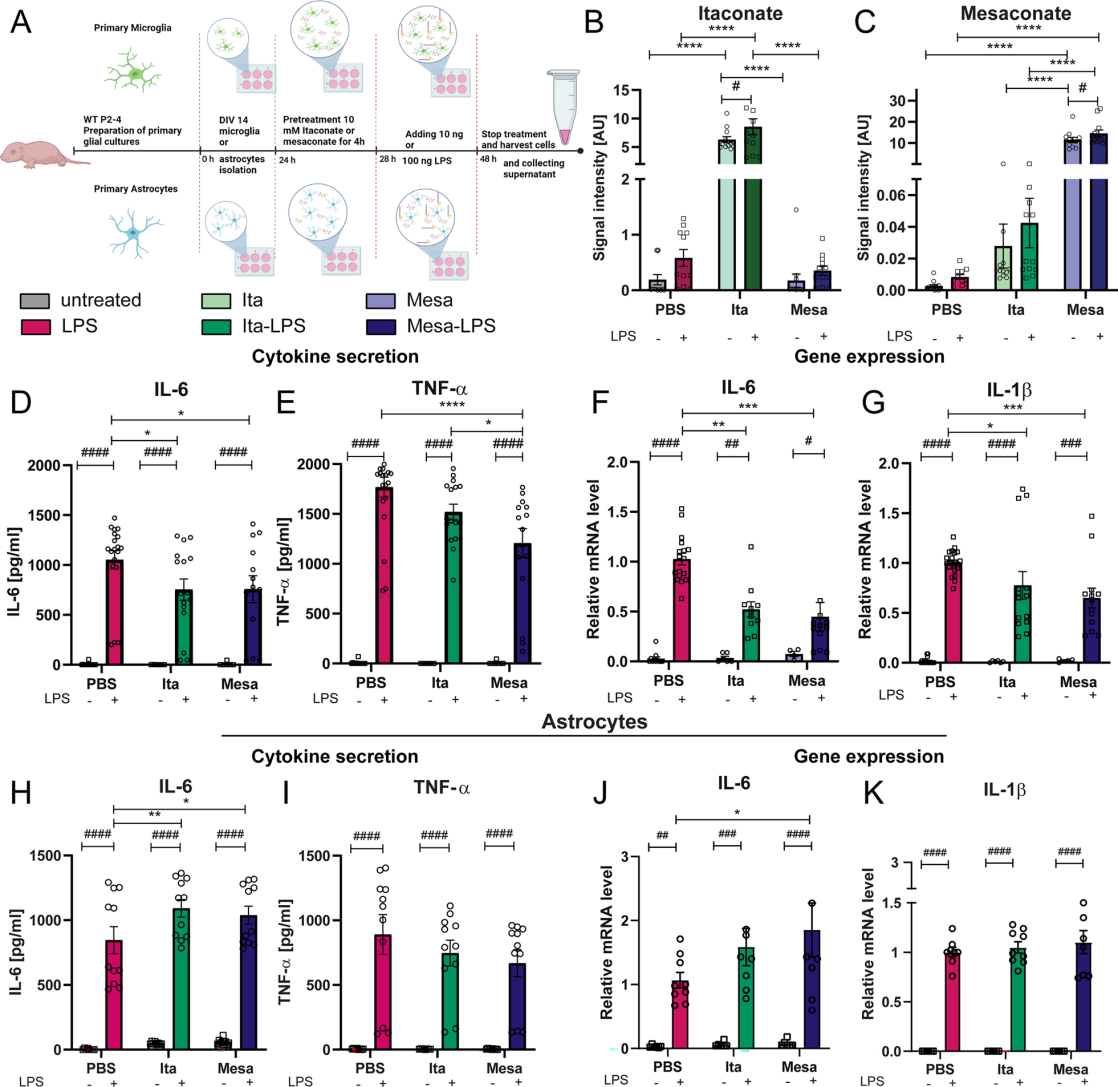
Pretreatment with itaconate or mesaconate dampen microglial but not astrocytic inflammatory response to LPS. **A** Experimental procedure of primary microglia and astrocytes pre-treated with itaconate and mesaconate followed by LPS stimulation (Created with BioRender.com). (**B**, **C**) Signal intensity of intracellular itaconate (**B**) and mesaconate (**C**) in primary microglia after either treated with itaconate or mesaconate for 24 h or additionally stimulated with LPS (10 ng/mL) after 4 h pretreatment. Cytokine secretion of (**D**) IL-6 and (**E**) TNF-α, as well as cytokine gene expression of (**F**) IL-6 and (**G**) IL-1β of primary microglia. Data are presented as mean ± SEM and were analyzed with a two-way ANOVA; */#p < 0.05, **/##p < 0.01, ***/###p < 0.001, ****/####p < 0.0001, [**B**, **C**: n = 11–12 biological replicates of 4 independent experiments. cytokine secretion: **D**: N = 2–5 per experimental group, n = 6–19, **E**: N = 2–4, n = 6–21; gene expression **F**: N = 2–4, n = 6–21, **G**: N = 2–4, n = 6–21. Astrocytes: cytokine secretion: **H**: N = 4 per experimental group, n = 8–11, **I**: N = 4, n = 8–11; gene expression **J**: N = 3, n = 6–9, K: N = 3, n = 6–9]

After demonstrating a dampened LPS-induced neuroinflammation in mice pretreated with itaconate or mesaconate, we aimed to identify the neuroinflammatory modulation of the two metabolites. Therefore, primary microglia- and primary astrocytes were prepared from neonatal mice and pretreated with itaconate or mesaconate before stimulation with LPS (Fig. 6A).

A four hour pretreatment of primary microglia with itaconate or mesaconate demonstrated an efficient uptake of the metabolites from the medium (Fig. 6, B: itaconate: untreated vs. Ita p < 0.0001, Mesa vs. Ita p < 0.0001, LPS vs. Ita-LPS p < 0.0001, Ita-LPS vs. Mesa-LPS p < 0.0001; C: mesaconate: untreated vs. Mesa p < 0.0001, Ita vs. Mesa p < 0.0001, LPS vs. Mesa-LPS p < 0.0001, Ita-LPS vs. Mesa-LPS p < 0.0001, Table 6). Moreover, when microglia were additionally treated with LPS (10 ng/mL), the intracellular metabolite levels of itaconate and mesaconate increased compared to control cells only treated with the metabolites without LPS stimulation (Fig. 6, B: Ita vs. Ita-LPS, p = 0.0137; C: Mesa vs. Mesa-LPS, p = 0.0131, Table 6). Notably, microglia pretreated with itaconate exhibited an increase in intracellular mesaconate levels, significantly less than that observed in microglia cells treated directly with mesaconate (Fig. 6C, Ita vs. Mesa p < 0.0001, Table 6). However, compared to cells that received no metabolite treatment, there was a slight increase in the level, although this increase did not achieve statistical significance. The increase in intracellular mesaconate level was only visible when cells were pretreated with itaconate. Microglia pretreated with mesaconate did not show an increase in the intracellular level of itaconate (Fig. 6B), which indicates a transformation of itaconate and mesaconate in a single-directional matter, as reported in macrophages [19]. Considering the evidence suggesting that itaconate might transform into mesaconate within microglia and based on the findings of He et al. that mesaconate is produced from itaconate in peripheral macrophages, we next aimed to know if this conversion also takes place in primary microglia (Suppl. 2) [19]. To study metabolic fluxes in LPS-stimulated primary microglia, we treated these cells with a [U-13C] glucose tracer, followed with analysis of the mass isotopomer distribution of downstream metabolites. If mesaconate originates from itaconate, its labeling pattern should mirror that of itaconate, albeit to a lesser degree, as demonstrated by He and colleagues in their study on peripheral macrophages [19]. Upon a twenty-four hour LPS stimulation, the highest fraction found were M1 mass isotopomers of itaconate, which represents molecules directly resulting from synthesized cis-aconitate, while the second highest fraction were the M3 isotopologues, which were derived from cis-aconitate synthesized in the second round of the TCA cycle (Suppl. 2A). A similar enrichment pattern was observed for mesaconate, whereas the isotopomer abundances of mesaconate were all slightly lower than those of itaconate (Suppl. 2A), in line with the results from He et al. [19].

**Table 6:** Significances Fig. 6

Figure 6B	Itaconate	F_treatment_(2,65) = 3–319, p = 0.0731
		F_treatment_(2,65) = 85.00, p < 0.0001
Figure 6C	Mesaconate	F_treatment_(2,65) = 164.5, p < 0.0001
		F_treatment_(2,65) = 2.166, p = 0.1459
Figure 6D	ELISA IL-6 Microglia	F_LPS-stimulation_ (1, 65) = 82.47, p < 0.0001
		F_treatment_(2, 65) = 1.330, p = 0.2716
Figure 6E	ELISA TNF-α Microglia	F_LPS-stimulation_ (1, 73) = 248.2, p < 0.0001
		F_treatment_ (2, 73) = 3.367, p < 0.0399
Figure 6F	Gene expression IL-6 Microglia	F_LPS-stimulation_ (1, 55) = 59.66, p < 0.0001
		F_treatment_(2, 55) = 59.66, p = 0.0055
Figure 6G	Gene expression IL-1β Microglia	F_LPS-stimulation_ (1, 69) = 107.2, p < 0.0001
		F_treatment_(2, 69) = 2.251, p = 0.1130
Figure 6H	ELISA IL-6 Astrocytes	F_LPS-stimulation_(1, 52) = 299.8, p < 0.0001
		F_treatment_(2, 52) = 2.693, p = 0.0771
Figure 6I	ELISA TNF-α Astrocytes	F_LPS-stimulation_(1, 52) = 87.77, p < 0.0001
		F_treatment_ (2, 52) = 0.6445, p = 0.5291
Figure 6J	Gene expression IL-6 Astrocytes	F_LPS-stimulation_ (1, 37) = 51.45, p < 0.0001
		F_treatment_(2, 37) = 1.494, p = 0.2376
Figure 6K	Gene expression IL-1β Astrocytes	F_LPS-stimulation_ (1, 37) = 383.1, p < 0.0001
		F_treatment_(2, 37) = 0.2987, p = 0.7436

He et al. further demonstrated that in peripheral macrophages mesaconate synthesis could also be attributable from glutamine via the TCA cycle and itaconate. To verify this in microglia, a [U-13C] glutamine tracer was applied to LPS-stimulated microglia (Suppl. 2B). Similar to peripheral macrophages [19], LPS-treated microglia showed a high abundance of M4 isotopomer of mesaconate and itaconate. Again, the mass isotopomer distribution of mesaconate was very similar to that of itaconate, but in lower abundance (Suppl. 2B). These findings suggest that mesaconate and itaconate are generated from the same pathway while mesaconate is in a later position than itaconate, as previously described in peripheral macrophages [19].

To verify that the observed decrease in microglial reactivity following in vivo LPS injection is due to a direct influence of itaconate or mesaconate on microglia, we proceeded to measure the release of pro-inflammatory cytokines in primary microglia stimulated with LPS, both with and without itaconate or mesaconate pretreatment. The LPS-stimulation led to an increased release of the pro-inflammatory cytokines IL-6 and TNF-α into the supernatant of primary microglial cultures, in comparison to untreated control groups, regardless if microglia were pretreated with itaconate or mesaconate (Fig. 6, untreated vs. LPS, D: IL-6 p < 0.0001, E: TNF-α p < 0.0001; Ita-untreated vs Ita-LPS, D: IL-6 p < 0.0001, E: TNF-α p < 0.0001; Mesa-untreated vs. Mesa-LPS, D: IL-6 p < 0.0001; E: TNF-α p < 0.0001, Table 6). Remarkably, the pretreatment with both metabolites diminished the release of pro-inflammatory cytokines. Specifically, the cells pretreated with mesaconate exhibited a significant decrease in both IL-6 and TNF-α while itaconate only significantly decreased the IL-6 level (Fig. 6, LPS vs. Mesa-LPS, D: IL-6 p = 0.0250; E: TNF-α p < 0.0001; LPS vs Ita-LPS, D: IL-6 p = 0.0164; E: TNF-α p = 0.0555, Table 6). These results are supported by the gene expression analysis of IL-1β and IL-6. The LPS-induced expression of IL-1β and IL-6 was reduced by both metabolites in a highly significant manner (Fig. 6, untreated vs. LPS, F: IL-6: p < 0.0001; G: IL-1β p < 0.0001, Ita-untreated vs. Ita-LPS, F: IL-6 p = 0.0011, G: IL-1β p < 0.0001, Mesa-untreated vs. Mesa-LPS, F: IL-6 p = 0.0245, G: IL-1β p = 0.0002, LPS vs Ita-LPS C: IL-6 p = 0.0024, D: IL-1β p = 0.0198; LPS vs Mesa-LPS, C: IL-6 p = 0.0004, D: IL-1β p = 0.0006, Table 6).

Considering the findings by Cordes et al., showing that itaconate treatment affects the metabolic program of astrocytes [27], we next investigated whether the pretreatment with itaconate or mesaconate also diminishes the inflammatory response in astrocytes. To this aim, we pretreated primary astrocytes, similarly to primary microglia, with itaconate or mesaconate, followed by LPS-exposure (Fig. 6H–K). Astrocytes stimulated with LPS showed a significant release of IL-6 and TNF-α into the supernatant compared to non-stimulated controls, regardless of pretreatment with itaconate and mesaconate (Fig. 6, untreated vs. LPS, H: IL-6 p < 0.0001, I: TNF-α p < 0.0001; Ita-untreated vs Ita-LPS, H: IL-6 p < 0.0001, I: TNF-α p < 0.0001; Mesa-untreated vs. Mesa-LPS, H: IL-6 p < 0.0001, I: TNF-α p < 0.0001, Table 6). Notably, following LPS stimulation, the levels of these cytokines were not decreased in the supernatant of astrocyte cultures pretreated with both itaconate and mesaconate, and even IL-6 was increased by both itaconate and mesaconate (Fig. 6H, LPS vs. Ita-LPS p = 0.0076, LPS vs. Mesa-LPS p = 0.0342, Table 6). Similarly, the gene-expression analysis revealed no reduction in LPS-stimulated *IL-1β* and *IL-6* expression by the pretreatment of both metabolites (Fig. 6, untreated vs. LPS, J: IL-6 p < 0.0001, K: IL-1β p < 0.0001, Ita-untreated vs. Ita-LPS, J IL-6 p = 0.0001, K: IL-1β p < 0.0001; Mesa-untreated vs. Mesa-LPS, J: IL-6 p < 0.0001, K: IL-1β p < 0.0001, LPS vs. Mesa-LPS, J: IL-6 p = 0.0177, Table 6).

Altogether, both itaconate and mesaconate mitigated the in vitro inflammatory response in LPS-stimulated microglia but not in astrocytes, therefore suggesting their beneficial effects observed in vivo attributable to the direct immuno-modulation in and by microglia.

### Impacts of *Irg1-*deficiency and the lack of endogenous itaconate synthesis on LPS-induced neuroinflammation

**Fig. 7:**
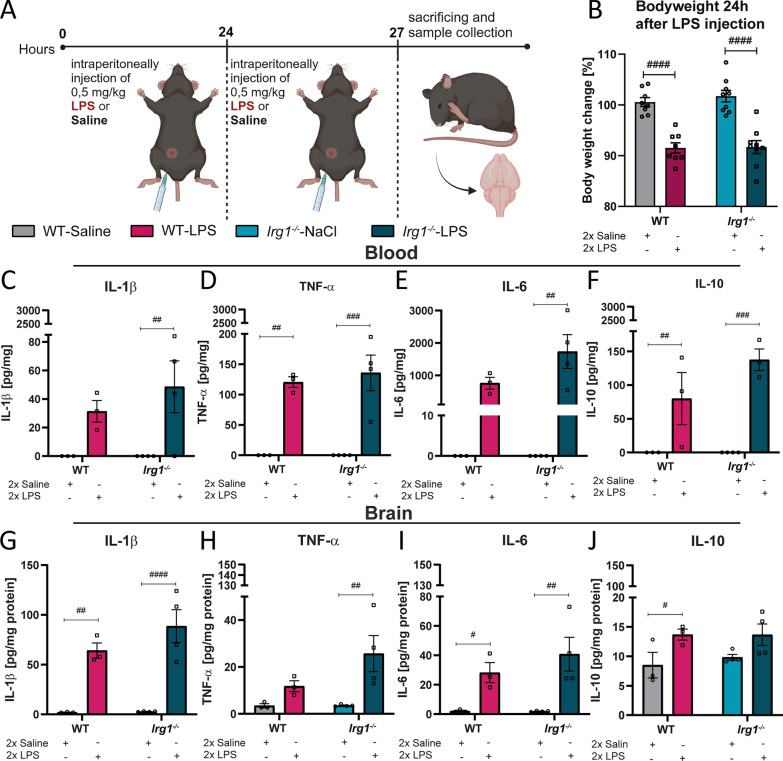
LPS triggers a strong inflammatory response in both WT- and *Irg1*^*−/−*^-mice. **A** Illustration of the injection protocol used in subsequent in vivo studies to compare the inflammatory respond between WT- and *Irg1*^−/−^-mice (Created with BioRender.com). **B** Bodyweight of the experimental groups twenty-four hours after the first LPS-injection. Cytokines in the blood (**C**–**F**) and brain (**G**–**J**) using ELISA. Data are presented as mean ± SEM and were analyzed with the two-way ANOVA followed by Fisher’s LSD test; *p < 0.05, ##p < 0.01, ###p < 0.001, ####p < 0.0001, [**B**: N (number of mice per group) = 8–9; **C**–**J**: N = 3–4]

Considering the fundamental roles of itaconate and mesaconate in the immune responses, we aimed to investigate how the absence of endogenous itaconate, resulted from *Irg1-*deficiency in *Irg1*^−/−^ mice, impacts LPS-induced neuroinflammatory response within the brain. Previous research indicates that endogenous itaconate plays a crucial role to restrict inflammatory responses, *Irg1*^*−/−*^ mice exhibit exacerbated disease outcomes compared WT mice capable of producing itaconate [47–51]. In a LPS-induced septic shock model, *Irg1*^−/−^ mice were subjected to more severe symptoms than the WT mice [52]. Kuo et al. discovered that *Irg1*^*−/−*^ mice, when subjected to stroke paradigms, suffered from significantly worse brain injuries [53]. These injuries were marked by larger cerebral infarcts, greater disruption of the blood–brain barrier, and heightened microglial activation, in comparison to their WT counterparts [53]. However, the impact of *Irg1-*deficiency on brain immunity remains poorly understood. To evaluate the function of IRG1 and its associated endogenous production of itaconate and mesaconate in LPS-induced neuroinflammation, both WT and *Irg1*^*−/−*^ mice received two i.p. LPS injections (0.5 mg/kg in a twenty-four-hour time interval) or saline solution as control (Fig. 7A). Subsequently, the inflammatory responses within the brains were analyzed three hours after the second injection of LPS and compared between the two genotypes. Twenty-four hours post-injection, the bodyweight of LPS-injected mice decreased in both *Irg1*^*−/−*^ and WT mice, in contrast to saline injected mice, with no significant influence of the genotype (Fig. 7B, WT-Saline vs. WT-LPS p < 0.0001; *Irg1*^*−/−*^-Saline vs. *Irg1*^*−/−*^-LPS p < 0.0001, Table 7). To investigate whether the absence of IRG1 influences the LPS-induced inflammatory responses, cytokine levels were measured in both blood serum as well as in brain homogenates by ELISA (Fig. 7C–F). As expected, the dual LPS injection induced secretion of IL-1β, TNF-α, IL-6, and IL-10 in WT mice, which was further strengthened in *Irg1*^*−/−*^ mice (Fig. 7, *Irg1*^*−/−*^ -Saline vs. *Irg1*^*−/−*^*-*LPS, C: IL-1β p = 0.0078, E: IL-6 p = 0.0019, D: TNF-α p = 0.0002; F: IL-10 p = 0.0005, Table 7). It is noteworthy that although *Irg1*^*−/−*^ showed slightly higher levels of pro-inflammatory mediators in the blood serum following LPS-stimulation, these differences were not statistically significant when compared to LPS-injected WT mice.

**Table 7:** Significances Fig. 7

Figure 7B	Bodyweight loss 24h after LPS		F_LPS-injection_ (1, 30) = 74.77 p < 0.0001
			F_genotype_ (1, 30) = 0.3624, p = 0.5517
Figure 7C	IL-1β Serum	F_LPS-stimulation_ (1, 10) = 12.76, p = 0.0051	
		F_genotype_ (1, 10) = 0.5936, p = 0.4588	
Figure 7D	TNF-α Serum	F_LPS-stimulation_ (1, 10) = 52.16, p < 0.0001	
		F_genotype_ (1, 10) = 0.1907, p = 0.6716	
Figure 7E	IL-6 Serum	F_LPS-stimulation_ (1, 10) = 15.40, p = 0.0051	
		F_genotype_ (1, 10) = 2.351, p = 0.1562	
Figure 7F	IL-10 Serum	F_LPS-stimulation_ (1, 10) = 32.38, p = 0.0003	
		F_genotype_ (1, 10) = 2.273, p = 0.1659	
Figure 7G	IL-1β Brain	F_LPS-injection_(1, 10) = 51.53, p < 0.0001	
		F_genotype_ (1, 10) = 1.500, p = 0.2487	
Figure 7H	TNF-α Brain	F_LPS-injection_ (1, 10) = 10.69, p = 0.084	
		F_genotype_ (1, 10) = 2.252, p = 0.1643	
Figure 7I	IL-6 Brain	F_LPS-injection_(1, 10) = 19.45, p = 0.0013	
		F_genotype_ (1, 10) = 0.7014, p = 0.4219	
Figure 7J	IL-10 Brain	F_LPS-injection_ (1, 10) = 9.122, p = 0.0129	
		F_genotype_ (1, 10) = 0.1843, p = 0.6768	

Analysis of cytokine levels in whole brain homogenates demonstrated that LPS-injection significantly elevated cytokine levels in both genotypes when compared to their respective saline controls (Fig. 7, WT-Saline vs. WT-LPS, G: IL-1β p = 0.0026, I: IL-6 p = 0.0410, J: IL-10 p = 0.0445; *Irg1*^*−/−*^ -Saline vs. *Irg1*^*−/−*^ -LPS, G: IL-1β p < 0.0001, I: IL-6 p = 0.0024, H: TNF-α p = 0.0046, Table 7).

Notably, LPS-induced secretion of IL-1β, IL-6 and TNF-α were more significantly increased in *Irg1*^−/−^ mice compared to WT mice. Taken together, our data indicate that the lack of endogenous itaconate production only had a minor effect on the inflammatory cytokine response triggered by LPS.

### Alterations in the endogenous synthesis of itaconate has no significant effect on LPS-induced microglial reactivity

**Fig. 8:**
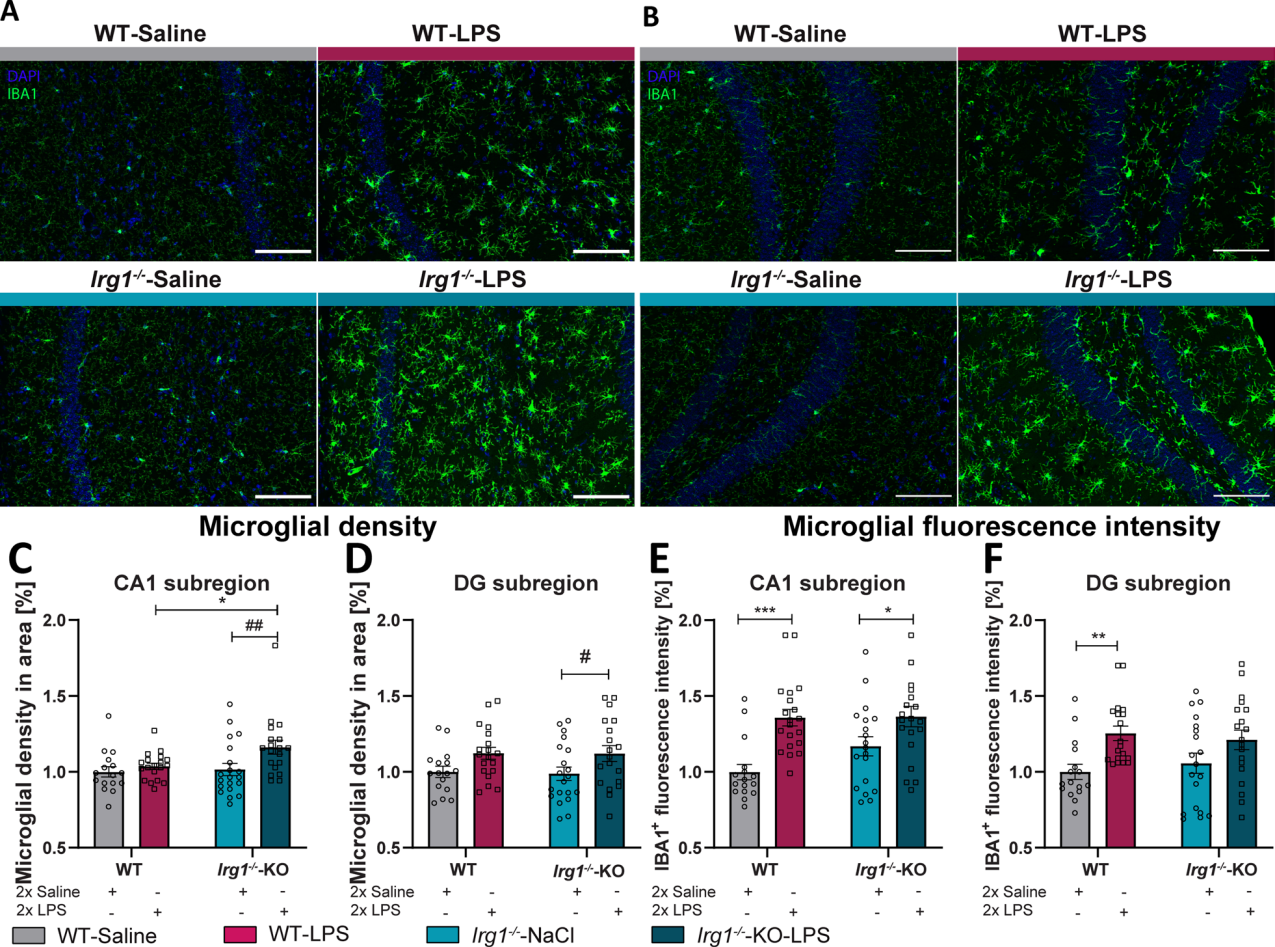
Silencing *Irg1* has no significant effect on IBA1 microglia reactivity in LPS-treated mice compared to WT. **A** Representative images from immunohistochemical staining for IBA1 and DAPI within the CA1 (**A**) and DG (**B**) across the experimental groups (magnification 20x, scale bar is 100 µm). Microglial density in the hippocampal CA1 (**C**) and DG (**D**). Fluorescence intensity of IBA1 in the CA1 (**E**) and DG (**F**). Data are presented as mean ± SEM and were analyzed with the two-way ANOVA followed by Fisher’s LSD test; */#p < 0.05, **/##p < 0.01, ***p < 0.001, [**C**, **D**: N (number of mice per group) = 4–5 per group, CA1 n (number of analyzed imaged per group) = 16–20, DG n = 16–20; **E**, **F**: N = 4–5 per group, CA1 n = 16–20, DG n = 16–20]

In vitro experiments, earlier described in this study, using primary microglia cultures of WT pretreated with itaconate before stimulated with LPS, demonstrated a reduced inflammatory phenotype (Fig. 6). Consequently, we investigated whether the absence of IRG1 and its respective lack of itaconate production affects the LPS-induced microglial reactivity in the hippocampus (Fig. 8A, B). LPS-injection resulted in a significantly increased number of IBA1^+^-cells within the hippocampal regions CA1 and DG of *Irg1*^*−/−*^ compared to those *Irg1*^*−/−*^ mice receiving saline (Fig. 8, *Irg1*^*−/−*^-saline vs. *Irg1*^*−/−*^-LPS, C: CA1 p = 0.0051; D: DG p = 0.0334, Table 8). Contrary, WT mice only showed modest increase microglial density in response to LPS when compared to saline-injected WT mice. Notably, a comparison of LPS-injected mice across both genotypes revealed a significant elevation in microglial density within the CA1 in *Irg1*^*−/−*^ mice. Moreover, post LPS-injection a significant increase in IBA1 fluorescence intensity was observed in the CA1 and DG of WT mice (Fig. 8, WT-Saline vs. WT-LPS, E: CA1 p = 0.0001, F: DG p = 0.0038, Table 8). In contrast, *Irg1*^*−/−*^ mice exhibited a significant enhancement in IBA1 fluorescence in the CA1 region and a notable, albeit non-significant, increase in the DG region (Fig. 8, *Irg1*^*−/−*^-saline vs. *Irg1*^*−/−*^-LPS, E: CA1 p = 0.0218, Table 8). While LPS administration induced an inflammatory microglial phenotype in mice, the absence of endogenous itaconate production, due to the deletion of *Irg1*, did not significantly affect this response. However, analysis of the microglial activation marker CD68 via immunofluorescence showed no significant elevation in CD68 fluorescence in the hippocampal subregions after LPS injection, compared to mice injected with saline neither in WT nor in *Irg1*^*−/−*^ mice (Suppl. 3A-D). Only in the CA1 subregion of WT mice a slight trend towards increased CD68 levels after LPS-injection was observed (Suppl. 3B, WT-Saline vs. WT-LPS p = 0.0675, Table 9). Additionally, the quantification of immunolabelled astrocytes via a GFAP staining revealed no significant elevation in GFAP fluorescence intensity between LPS- and saline-injected mice of either genotype (Suppl. 4A-D). Only a slightly higher GFAP expression was observed in both the CA1 and DG subregions of the hippocampus of WT mice after LPS-injection, and in the CA1 subregion of *Irg1*^−/−^ mice (Suppl. 4B,D). Remarkably, although LPS injection leads to heightened density and reactivity of microglial cells within the hippocampus, the absence of IRG1 and the consequent lack of endogenous itaconate production did not significantly influence the observed increases in microglial density and reactivity triggered by LPS.

**Table 8:** Significances Fig. 8

Figure 8C	IBA1 cell density CA1	F_LPS-injection_ (1, 70) = 8.224, p = 0.0055
		F_genotype_ (1, 70) = 3.662, p = 0.0597
Figure 8D	IBA1 cell density DG	F_LPS-injection_ (1, 70) = 6.132, p = 0.0157
		F_genotype_ (1, 70) = 0.02670, p = 0.8707
Figure 8E	IBA1 fluorescence intensity CA1	F_LPS-injection_ (1, 71) = 21.09, p < 0.0001
		F_genotype_ (1, 71) = 2.156, p = 0.1465
Figure 8F	IBA1 fluorescence intensity DG	F_LPS-injection_ (1, 69) = 12.17, p = 0.0008
		F_LPS-injection_ (1, 69) = 0.01242, p = 0.9116

**Table 9:** Supplementary 3 and 4

Suppl. 3B	CD68 fluorescence intensity CA1	F_LPS-injection_ (1, 71) = 4.305, p = 0.0416
		F_genotype_ (1, 71) = 3.407, p = 0.0691
Suppl. 3D	CD68 fluorescence intensity DG	F_LPS-injection_ (1, 72) = 0.06944, p = 0.7929
		F_genotype_ (1, 72) = 0.1633, p = 0.6874
Suppl. 4B	GFAP fluorescence CA1	F_LPS-injection_ (1, 71) = 2.213, p = 0.1413
		F_genotype_ (1, 71) = 4.828, p = 0.0313
Suppl. 4D	GFAP fluorescence DG	F_LPS-injection_ (1, 68) = 0.3305, p = 0.5672
		F_genotype_ (1, 68) = 1.031, p = 0.3135

### Both WT and *Irg1*^−/−^ mice exhibited LPS-induced impairments in synaptic transmission and plasticity

**Fig. 9:**
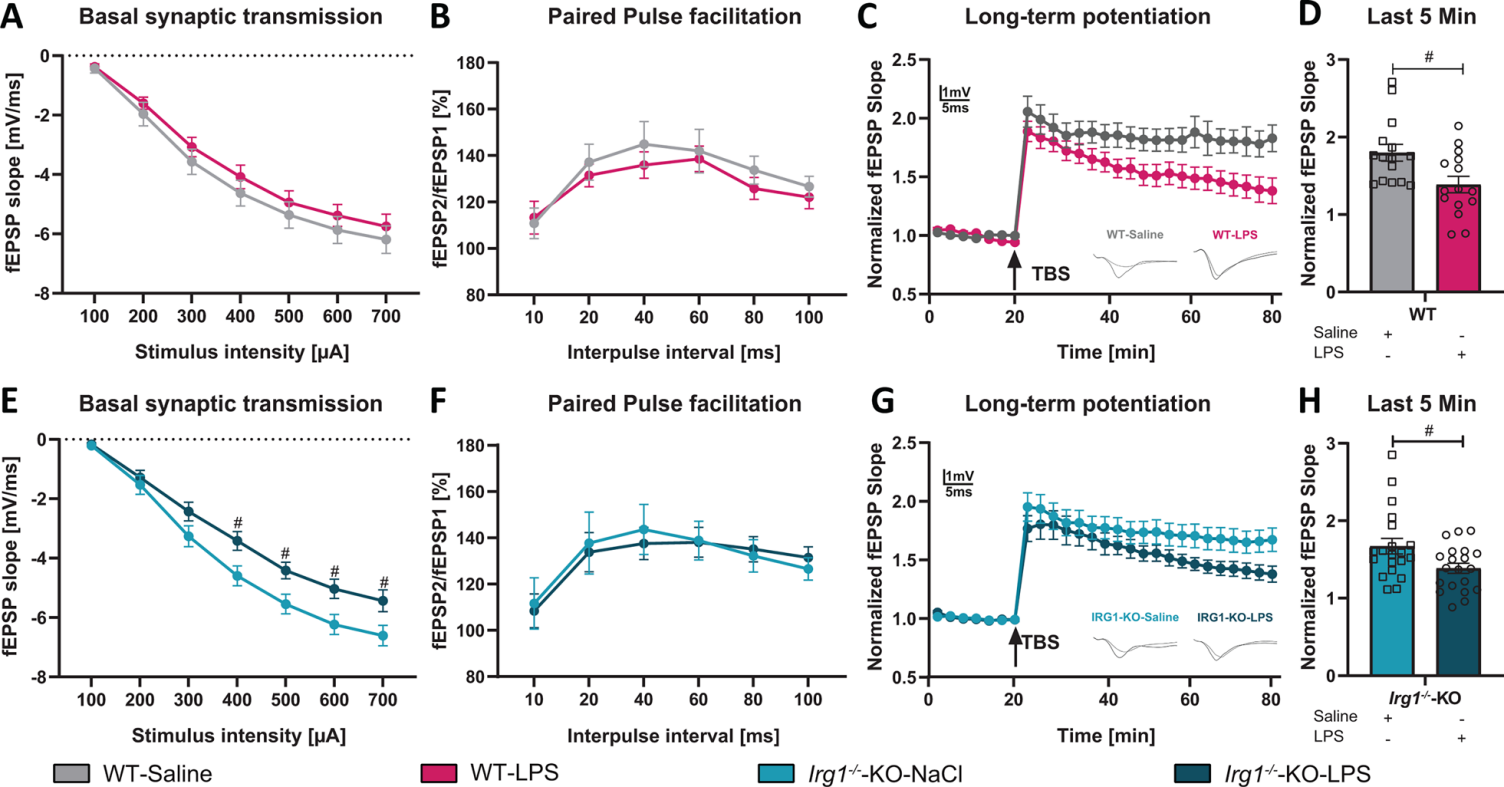
*Irg1*^*−/−*^-mice do not show an effect on LTP induced by LPS compared to WT-mice. **A** Input–output curves of field excitatory postsynaptic potential (fEPSP) slopes in acute hippocampal slices of WT- (**A**) and *Irg1*^*−/−*^*-mice *(**E**). Paired-pulse facilitation (PPF) of the fEPSP slopes plotted as a response to the second stimulation over the first one at different interpulse intervals (10, 20, 40, 60, 80, and 100 ms) in hippocampal slices of WT- (**B**) and *Irg1*^*−/−*^*-mice* (**F**). LTP was measured in hippocampal acute slices of WT- (**C**) and *Irg1*^*−/−*^*-mice*. (**G**) LTP maintenance (T 75–80 min) was assessed in hippocampal acute slices of WT- (**D**) and *Irg1*^*−/−*^*-*mice (**H**). Data are presented as mean ± SEM and were analyzed with the repeated measures two-way ANOVA followed by Fisher’s LSD test (**A**, **B**, **C**, **E**, **F**, **G**) or (**D**, **H**) unpaired t-test.; #p < 0.05, [N (number of mice per group) = 3–4, n (number of analyzed hippocampal slices per group) = 15–20]

To investigate the role of *Irg1-*deletion in LPS-induced neuroinflammation in greater detail, we examined the effect of *Irg1-*deletion and its respective loss of endogenous itaconate production, on activity-dependent synaptic plasticity. Specifically, we assessed whether the degree of impairment in synaptic plasticity induced by LPS varies between WT and *Irg1*^*−/−*^ mice. To this end, electrophysiological assessments was conducted (Fig. 9). Analyzing basal synaptic transmission in WT mice, no significant differences were observed between those injected with LPS and those with saline (Fig. 9A, E). However, in *Irg1*^*−/−*^ mice LPS injection resulted in a reduced fEPSP slope at stimulus intensities of 400, 500, 600, and 700 μA compared to *Irg1*^*−/−*^ mice injected with saline, indicating a notable impairment in synaptic transmission (Fig. 9E, *Irg1*^*−/−*^-saline vs. *Irg1*^*−/−*^-LPS: 400 μA p = 0.0146, 500 μA p = 0.0132, 600 μA p = 0.0146, 700 μA p = 0.0248, Table 10). Subsequently, we assessed short-term synaptic plasticity at the Schaffer collateral-CA1 synapse PPF (Fig. 9B, F). No notable differences in PPF were observed between treatments in either WT or *Irg1*^*−/−*^ mice, indicating that presynaptic function remained largely unaffected by LPS injection in both genotypes. Lastly, the long-term activity-dependent synaptic plasticity was examined (Fig. 9C, D, G, H). As before, LTP was induced at the Schaffer collateral CA3 to CA1 pathway using TBS after a 20-min period of stable baseline recording (Fig. 9C, G). Our findings showed that LTP was significantly impaired in the hippocampus of mice following LPS injection compared to saline-injected controls, regardless of genotype. This impairment extended to both genotypes during the maintenance phase of LTP post-LPS injection (Fig. 9, WT, D: p = 0.0119; *Irg1*^*−/−*^ H: p = 0.0228, Table 10).

**Table 10:** Fig. 9

Figure 9A	fEPSP WT	F_treatment_ (1, 28) = 0.7518, p = 0.3933
		F_stimulus_ (1.856, 51.95) = 238.3, p < 0.0001
Figure 9B	PPF WT	F_treatment_ (1, 28) = 0.3322, p = 0.5690
		F_stimulus_ (2.825, 79.10) = 17.65, p < 0.0001
Figure 9E	fEPSP IRG1-KO	F_treatment_ (1, 38) = 5.572, p = 0.0235
		F_stimulus_ (2.217, 84.26) = 288.5, p < 0.0001
Figure 9F	PPF IRG1-KO	F_treatment_ (1, 38) = 0.009346, p = 0.9235
		F_stimulus_ (2.106, 80.01) = 17.86, p < 0.0001

Overall, our findings indicate that the deletion of the *Irg1* gene and the respective absence of endogenous itaconate do not significantly influence the LPS-induced impairment in activity-dependent synaptic plasticity within the hippocampus.

## Discussion

This work aimed to identify novel therapeutic agents that could mitigate neuroinflammatory processes, thereby reducing their profound long-term effects on neuronal health. We focused particularly on endogenous compounds, which are naturally synthesized by macrophages and thus have higher biocompatibility and tolerability. Itaconate and mesaconate, two prominent immunomodulatory agents, may not only be therapeutic candidates for infection-induced inflammation in the periphery [19], but were also presented in this work for their potential to attenuate neuroinflammatory processes triggered by bacterial endotoxins. The results of this study now clearly indicate that the TCA metabolites itaconate and mesaconate can ameliorate bacterial endotoxin effects on the release of pro-inflammatory cytokines, microglial reactivity and can rescue impaired synaptic plasticity in the brain.

This is important to elucidate, since neuroinflammatory responses are known to have long-lasting deleterious effects on brain, in particular on the hippocampus, an especially vulnerable brain region [54–58]. Neuroinflammation can eventually impair cognitive functions such as learning and memory processes well beyond the initial phase of sepsis or viral infection [6, 29]. Additionally, neuroinflammation has been linked to the progression of various neuropathological conditions, including neurodegenerative diseases like Alzheimer’s disease (AD) [14, 15, 59–61]. Despite extensive research, an effective drug treatment remains elusive.

The evidence obtained here suggests that the endogenous production of itaconate and mesaconate may not be sufficient to attenuate neuroinflammation, as shown by the comparison of WT and *Irg1*^*−/−*^ mice exposed to LPS, while additional exogenous administration of these immunomodulatory metabolites may be more effective via cumulative effects.

The dosage of exogenous itaconate and mesaconate (250 mg/kg body weight) used in this study was chosen based on findings by He et al., which showed that mice treated with these metabolites prior to administrations of a lethal dose of LPS experienced extended survival rate compared to untreated mice [19]. In addition, mice pretreated with itaconate or mesaconate and injected with LPS showed less severe symptoms and a lower drop in body temperature [19]. The LPS dose for our study, 0.5 mg/kg body weight, was chosen in line with our previous study, identifying the dose effective in triggering neuroinflammatory symptoms through a dual LPS injection [7, 37].

The release of pro-inflammatory cytokines, which is a crucial element in the signaling and initiation of inflammatory responses [62–64], showed a significant decrease in the release and production of IL-1β, IL-6 and TNF-α in the brain when mice were pretreated with itaconate or mesaconate prior to LPS injection. This finding suggests that the two metabolites may attenuate a critical aspect in the initiation of a neuroinflammatory responses. Additionally, itaconate and mesaconate selectively decreased IL-1β levels in blood serum, which may underscore the attenuated general disease-like symptoms in the LPS-exposed mice. Furthermore, IL-1β injections into rodent brains activates astrocytes and microglia cells accordingly as earlier studies have shown [65], highlighting the role of IL-1β in contributing to neuroinflammatory processes and emphasizing its suitability as a target for neuroinflammation therapy. These findings are along to the previous investigations that showed ester of itaconate by promoting the expression of cyclic AMP-dependent transcription factor (ATF3) in mouse macrophages and human blood monocytes can prevent LPS-induced expression of IκBζ protein and IL-6 [66] which may also be the case in microglia in this scenario. In addition, dimethyl itaconate was shown to protect against LPS-induced pro-inflammatory mediators release in mice by activating MAPKs and Nrf2 and inhibiting NF-kB signaling pathways [67]. Future studies can identify the effects of metabolites in modulating NF-kB and IκBζ as underlying mechanistic signaling pathways. In another study, itaconate and its two isomers, mesaconate and citraconate, were found to decrease the phosphorylation levels of Signal Transducer and Activator of Transcription 1 (STAT1) and attenuate canonical type I IFN signaling [68]. Therefore, these signaling pathways may be involved in the immunomodulatory effects of the metabolites on microglial cells but need to be investigated in future studies to reveal itaconates and mesaconates signaling pathways in microglia. Conversely, we did not observe changes in levels of the anti-inflammatory cytokine IL-10, which actually slightly increased in mice pretreated with mesaconate prior to LPS injection compared to those injected with LPS alone. IL-10 is known for its strong anti-inflammatory properties [69]. It modulates tissue repair after an inflammatory insult. LPS can upregulate microglial IL-10 release both in vitro and in vivo, probably due to the stimulation of endogenous repair mechanisms in preparation for the clearance of the acute insult [70, 71]. Our finding in this respect is remarkable, since it suggests that the anti-inflammatory function of itaconate and mesaconate in the brain may be mediated via the downregulation of pro-inflammatory cytokines, and not via an increased production of anti-inflammatory cytokines.

Peritoneal injections of LPS have been shown to enhance microglial reactivity in the hippocampus [7, 42], therefore we intended here to elucidate the potential positive role of itaconate and mesaconate on microglial reactivity using as readouts cell density, IBA1 fluorescence intensity and the levels of the microglial activation marker CD68 in subregions of the hippocampus. Only itaconate prevented the LPS-induced increase in microglia density. Moreover, previous studies have demonstrated that LPS elevates IBA1 expression [41, 72]. Of note, pretreatment with both itaconate and mesaconate significantly reduced the fluorescence intensity of IBA1 immunostaining in the hippocampus following LPS injection compared to mice pretreated with PBS. Elevated microglial density and IBA1 expression levels are indicative of both increased microglial reactivity and consequently neuroinflammation triggered by LPS injection [42]. Considering the different effects of itaconate and mesaconate on microglia density and IBA1 expression in the hippocampus, it is possible that pretreatment with these metabolites modulates microglial reactivity differently and in co-supporting way. Mesaconate could attenuate aspects of the pro-inflammatory response, as shown by decreased IBA1 expression, while itaconate could prevent microglial proliferation in addition to attenuating pro-inflammatory responses. Previously, itaconate was shown to play a complex role in influencing macrophage polarization, suggesting a possible influence on macrophage phenotype [73, 74]. It is reasonable to assume that both itaconate and mesaconate regulate the inflammatory state of microglia, the macrophages residing in the brain, even though they appear to function differently. However, this hypothesis needs to be investigated by further studies analyzing the effect of itaconate and mesaconate on microglial polarization possibly leading to different functions and properties. In this respect it is noteworthy, that He et al. have shown that the two metabolites have different effects on cellular respiration and metabolic changes in macrophages [19]. Although both itaconate and mesaconate possess immunomodulatory potential, they seem to interact with cellular metabolic pathways in distinct ways [19], which might lead to the different regulatory states of the microglia. It was demonstrated that itaconate, but not mesaconate, inhibited Succinate dehydrogenase (SDH) in macrophages, while mesaconate treatment did not [19]. Furthermore, although mesaconate attenuated glycolytic activity similarly to itaconate, only itaconate was able to suppress TCA activity and cellular respiration [19]. Therefore, there are some differences in the mechanisms of action of these two metabolites on cells. It is therefore plausible that they also have different effects on the metabolic status of microglia, which may ultimately affect their functions and reactivity, a phenomenon that requires further investigation in future studies. This is even more remarkable considering that there are only minor differences between itaconate and mesaconate, both just differ due to the different position of a single proton and the placement of the double bond.

Recent research suggests that the categorization of the activation states of microglia, as opposed to peripheral macrophages, is more complex than previously assumed: The traditional binary categorization of microglia into “M1—classically activated, pro-inflammatory” and “M2—alternatively activated, anti-inflammatory/tissue repairing” is now considered simplistic [75]. It is more likely that microglia exhibit a spectrum of functional and morphological phenotypes that cannot be reduced to two categories (M1/M2 –reactive/not reactive). It may be that microglia from itaconate- and mesaconate-pretreated mice injected with LPS have adopted different stages of reactivity that differentially modulate microglial proliferation and reactivity. To gain a more comprehensive understanding of how pretreatment with itaconate and mesaconate may modulate LPS-induced microglial reactivity, further research involving morphological studies, gene expression profiling, and functional assessments may be beneficial.

LPS injection resulted in increased expression of CD68, a microglial activation marker, throughout the brain of mice pretreated with control PBS, indicating microglial reactivity in response to inflammation [76, 77]. Conversely, mice pretreated with itaconate and mesaconate exhibited a non-significant increase in CD68 levels as determined by FACS analysis. However, immunohistochemical examination of CD68 expression in hippocampal microglial cells did not reveal this effect very clearly. This finding emphasizes a modulation of microglial reactivity in mice pretreated with itaconate and mesaconate, which could be even more pronounced in other brain regions besides the hippocampus.

Part of the brains immune response in addition to microglia are astrocytes. However, when we quantified astrogliosis by analyzing GFAP fluorescence intensity our results indicate that the dose of LPS used in this study was not sufficient to induce severe astrogliosis, as LPS did not result in a significant increase in fluorescence intensity compared to corresponding saline injected controls. These results are consistent with other studies, e.g. Norden et al. came to similar conclusions and observed an increase in IBA1 expression twenty-four hours after LPS injection (0.33 mg/kg i.p. LPS), but no change in GFAP fluorescence at such a low dose of LPS, despite an increase in astrocytic cytokine profiles [42]. Further evidence along this line was presented by Wendeln et al., which observed that the number of GFAP^+^ astrocytes increased only after three low-dose LPS injections (0.5 mg/kg) [37]. Furthermore, Kang et al. reported increased GFAP fluorescence intensity following five LPS injections (250 µg/kg) [78]. However, it is noteworthy that in our study LPS-injected mice without pretreatment exhibited a more pronounced tendency for elevated GFAP expression compared to the corresponding controls.

Beyond the in vivo studies, in vitro investigations using isolated microglia and astrocyte cultures were carried out to identify the specific cell type through which the observed anti-inflammatory effects of metabolite treatment are mediated. Microglia pretreated with itaconate or mesaconate, but not astrocytes, showed a reduction in pro-inflammatory cytokines following LPS stimulation. This suggest that the anti-inflammatory effects observed in vivo are primarily mediated by the modulation of microglial function, whereas astrocytes may play a less important role in initiating the immunoregulatory effects of itaconate and mesaconate within the brain. Considering that activated microglial cells have the potential to induce astrocytic reactivity [11], the slight increase in GFAP immunostaining in the LPS-injected mice pretreated with itaconate and mesaconate compared to the mice receiving LPS alone could be attributed to reduced microglial activation induced by the metabolites.

Intraperitoneal injection of LPS has been shown to induce neuroinflammation leading to structural and functional changes in neuronal cells, particularly impairing cognitive function [6, 7, 79, 80]. In line with previous findings, the results here further supported the notion that mice injected with LPS exhibited impaired long-term potentiation (LTP) at CA3-CA1 synapses in the hippocampus. This impairment in LTP was rescued when mice were administered itaconate or mesaconate prior to LPS injection, supporting the potential of the two metabolites to prevent the consequences of neuroinflammation on synaptic plasticity. Previous reports suggested that inflammatory mediators play a role in impairing neuronal synaptic transmission and plasticity [81–83]. The detrimental effect of LPS on synaptic plasticity is attributed in part to the activation of pro-inflammatory cytokines such as IL-1β [80, 84–86]—a cytokine that in this study showed the most significant reduction in brain levels in LPS-injected mice when pretreated with itaconate or mesaconate compared to mice injected with LPS alone. While previous studies indicate that a low basal level of IL-1β is essential for the proper regulation of synaptic plasticity, excessively elevated levels of this cytokine can negatively affect synaptic transmission [81]. In addition, IL-1β has been found to impair signaling from N-methyl-d-aspartate (NMDA) and α-amino-3-hydroxy-5-methyl-isoxazole-4-propionic acid (AMPA) glutamate receptors [87, 88], although the exact regulatory mechanism of the metabolites in this scenario remains unclear [81].

In a next step of the study to determine the importance of endogenous itaconate levels in attenuating neuroinflammatory processes and adverse sequelae in vivo, we injected *Irg1*-deficient mice (*Irg1*^−/−^) with LPS. The *Irg1* gene encodes the enzyme IRG1, which catalyzes the synthesis of itaconate [20]. Previous studies have shown that *Irg1*^−/−^mice exhibit an enhanced inflammatory response and a more severe disease phenotype compared to WT mice with endogenous itaconate production [47–51]. In a model of septic shock, mice administered a single injection of LPS exhibited stronger symptoms of sepsis compared to their WT counterparts [52]. Although the effects of Irg1 deficiency on the peripheral immune response are well documented, there are few studies looking at the effects on the brain. Nevertheless, Kuo et al. et a discovered that *Irg1*^−/−^ mice suffered more severe brain injury after stroke [53]. In addition, Daniels et al. showed that mice lacking *Irg1* were more susceptible to infection by neurotropic flaviviruses, including Zika virus, than WT mice, with *Irg1*^−/−^ mice having a higher viral load [89]. Despite these findings, the role of Irg1 deficiency in brain-specific inflammation is less explored. Here, *Irg1*^−/−^ mice showed only slightly higher susceptibility to LPS injections compared to WT mice, with both genotypes experiencing similar body weight loss after peripheral LPS administration. Of note, pro-inflammatory mediators in the blood and brain of *Irg1*^−/−^ mice were slightly elevated compared to WT mice, although not significantly. Contrary to expectations based on previous studies, such as Yang et al., which reported significant differences in body weight loss and higher cytokine levels in *Irg1*^−/−^ mice, these results could not be replicated in this study, possibly due to variations in LPS dosage, timing of blood analysis or number of LPS injections [52]. In addition, the number of LPS injections, using a double injection strategy in the current study, has been shown to induce a strong stimulation of cytokines in the brain, but could induce immune tolerance in the periphery, reflected by altered cytokine levels in the blood, possibly explaining the observed results [37].

Analysis of microglial cells also revealed only minor changes between the two genotypes, with microglial density being higher in LPS-injected *Irg1*^−/−^ mice than in WT mice injected with LPS. However, there were no significant changes in the fluorescence intensity of IBA1 and CD68 immunostaining. Together with the slight increase in inflammatory mediators in *Irg1*^−/−^ mice compared to WT, this suggests that the absence of IRG1 has a limited effect on LPS-induced neuroinflammation. Furthermore, no changes were observed in astrocytes with respect to the intensity of GFAP immunofluorescence. When activity-dependent synaptic plasticity was examined in the hippocampus of both WT and *Irg1*^−/−^ mice, LTP was found to be impaired after two peripheral LPS injections. However, the extent of this LPS-induced impairment was not significantly different between *Irg1*^−/−^ and WT mice, suggesting that the impairment was not enhanced by the absence of IRG1 or, by extension, by the absence of endogenous itaconate. Overall, our results suggest that deletion of *Irg1* does not significantly affect the neuroinflammatory response to a double injection of a low dose of LPS. This contradicts the original hypothesis that the absence of endogenous itaconate production in *Irg1*^−/−^ mice would exacerbate neuroinflammation. Previous research has emphasized the role of IRG1 in itaconate production due to its immunomodulatory effects via multiple pathways. However, the functions of IRG1 go beyond immunomodulation [78, 79]. Li et al. pointed out the involvement of IRG1 in oxidative stress and found increased ROS levels in immunostimulated macrophages [79]. IRG1 is also associated with antigen processing by enhancing MHC class I molecules (MHC1) functionality and exhibiting antiviral properties via mechanisms that are not yet fully understood [80]. In addition, He et al. have shown that administration of itaconate and mesaconate, which are endogenously synthesized by IRG1, can increase the levels of IFN-β and CXCL10, highlighting the antiviral effects of these metabolites [19].

However, Wu et al. pointed out that the actions of IRG1 not only protect against inflammation but can also exacerbate tissue damage under certain conditions [78]. Thus, increased *Irg1* mRNA levels in macrophages infected with *V. Leishmania* facilitated parasite growth and survival [81], and *Irg1* expression was associated with lung damage during respiratory syncytial virus infection [82]. Furthermore, IRG1-driven itaconate production promotes vesicular stomatitis virus replication, with *Irg1*^−/−^ mice exhibiting reduced infiltration of inflammatory cells in the lung [83]. In addition, IRG1 has been associated with tumorigenesis, and knocking down *Irg1* can reduce tumor growth [90]. These results emphasize the complex role of IRG1 in promoting and inhibiting disease processes. They suggest that the absence of IRG1 does not consistently lead to worsened outcomes and emphasize the need for further research into its diverse biological effects.

Overall, our study focused on the acute phase of infection after LPS injection in order to determine how TCA metabolites might dampen the initiation of inflammation. Not within the scope of our study, was whether exogenously administered itaconate and mesaconate can attenuate the long-term effects of inflammatory processes induced by LPS, such as memory deficits and the negative neuronal effects triggered by LPS [6, 84, 85]. These could include behavioral assessments in the experimental groups used in this work to investigate how these compounds affect memory formation impaired by neuroinflammatory events as well as long-term morphological changes in neurons.

Considering that microglia play a crucial role in maintaining the balance and proper function of the CNS during development and adulthood, there is growing evidence that hyperreactivity and dysfunction of microglia in conjunction with their excessive release of inflammatory mediators can lead to neurotoxic outcomes in various neurological and neurodegenerative diseases [86]. Therefore, modulation of microglia reactivity is a potential therapeutic strategy to treat a number of neurological and neurodegenerative diseases characterized by neuroinflammation. As endogenous compounds, as the TCA metabolites itaconate and mesaconate are particularly interesting due to their biocompatibility and tolerability, which could also make them suitable for long-term treatment. However, to verify these advantages, thorough long-term studies are needed to confirm that prolonged administration has no detrimental effects. In addition, research into less invasive delivery methods than injections, such as oral administration via drinking water or food, could make these compounds suitable for oral therapies. A remarkable point in this study is the possibility that the observed effect of itaconate and mesaconate on neuroinflammation may be an indirect effect of them in influencing peripheral inflammation rather than a direct effect inside the CNS. Although the study cannot definitively rule out this possibility, the finding that itaconate levels in the brain remained elevated fifty-one hours after the last injection suggests the ability of itaconate to cross the blood–brain barrier (BBB) and be taken up by CNS cells. This highlights further its therapeutic potential for the treatment of neurological diseases, since this indicated a possible direct effect on microglia cells. However, the pharmacokinetic details of itaconate and mesaconate need to be investigated in future studies to understand the role of the two metabolites in modulating the immune response. However, it is important to note that despite the unknown and perhaps short half-life, itaconate and mesaconate appear to induce significant intracellular signaling cascades leading to their immunoregulatory functions, which need to be discovered by future studies. Moreover, the metabolites here were administered prior to LPS injection, mainly to demonstrate their preventive importance. It would be of interest to investigate whether post-treatment with itaconate and mesaconate could also attenuate the pre-existing neuroinflammatory consequences. However, to clarify this hypothesis, the timing of administration of the metabolite is very important, as the acute phase of immune responses triggered by LPS is very short and prompt. In addition, future studies need to clarify whether the findings related to LPS can be extrapolated to sterile causes of disease, as the pathogenic neuroinflammatory responses differ considerably from those caused by sterile insults such as proteinopathies or trauma. The path from laboratory research to clinical translation is complex and multi-layered. However, this work lays an important foundation and highlights the need for further research to fully exploit the therapeutic potential of itaconate and mesaconate in the fight against neuroinflammatory brain diseases.

## Conclusion

This study investigated the effects of itaconate and mesaconate, derived from the metabolites of the TCA cycle, on neuroinflammation in a mouse model of septic shock induced by i.p. injection of LPS. The results of this study showed that pretreatment with itaconate or mesaconate significantly reduced the levels of key pro-inflammatory cytokines in the brain. Importantly, these metabolites significantly attenuated the excessive microglial reactivity induced by LPS. Moreover, the protective effects of these metabolites on neuroinflammation-induced damage were able to reverse the impairment of synaptic plasticity and maintain the density of dendritic spines after LPS exposure. Overall, this study highlights the potential therapeutic value of itaconate and mesaconate in the treatment of neuroinflammatory diseases and emphasizes their important role in protecting neuronal structure and function. Due to their endogenous origin and production, which generally leads to high tolerance, these metabolites could be of great importance as therapeutic strategies in neuroinflammatory brain diseases.

### Supplementary Information

Supplementary Material 1.

## Data Availability

Data is provided within the manuscript or supplementary information files. The datasets used during the current study are available from the corresponding author upon reasonable request.

## Abbreviations

4-OI: 4-Octyl itaconate
ACOD1: Aconitate decarboxylase 1
ACSF: Artificial cerebrospinal fluid
AD: Alzheimer's disease
AMPA: α-Amino-3-hydroxy-5-methyl-isoxazole-4-propionic acid
BBB: Blood–brain barrier
BDNF: Brain-derived neurotrophic factor
C1q: Complement component 1q
CA: Cornu Ammonis
CD68: Cluster of differentiation 68
CNS: Central nervous system
CXCL10: C-X-C motif chemokine ligand 10
DG: Dentate gyrus
DMI: Dimethyl itaconate
ELISA: Enzyme-linked immune sorbent assa
FACS: Fluorescence-activated cell sorting
fEPSPs: Field excitatory postsynaptic potentials
GC–MS: Gas chromatography–mass spectrometry
GFAP: Glial fibrillary acidic protein
i.p.: Intraperitoneal
IBA1: Ionized calcium binding adaptor molecule-1
IL: Interleukin
IFN: Interferon
IRG1: Immune-responsive gene 1
Irg1-/-: Irg1-deficient mice
LPS: Lipopolysaccharide
LTP: Long-term potentiation
NMDA: N-methyl-d-aspartate
NO: Nitric oxide
NRF2: Nuclear factor erythroid-2-related factor 2
PBS: Phosphate buffered saline
PD: Parkinson’s disease
PFA: Paraformaldehyde
PGE2: Prostaglandin E2
PPF: Paired pulse facilitation
ROS: Reactive oxygen species
TBS: Theta-burst stimulation
TCA: Tricarboxylic acid cycle
WT: Wild-type
