# Supplementary material for: The potential therapeutic role of itaconate and mesaconate on the detrimental effects of LPS-induced neuroinflammation in the brain
Source: J Neuroinflammation. 2024 Aug 20;21:207. doi: 10.1186/s12974-024-03188-3 (PMC11337794; doi:10.1186/s12974-024-03188-3)
Supplement: Supplementary file 1 — Supplementary Material 1. [file 12974_2024_3188_MOESM1_ESM.docx]

**Supplementary material**
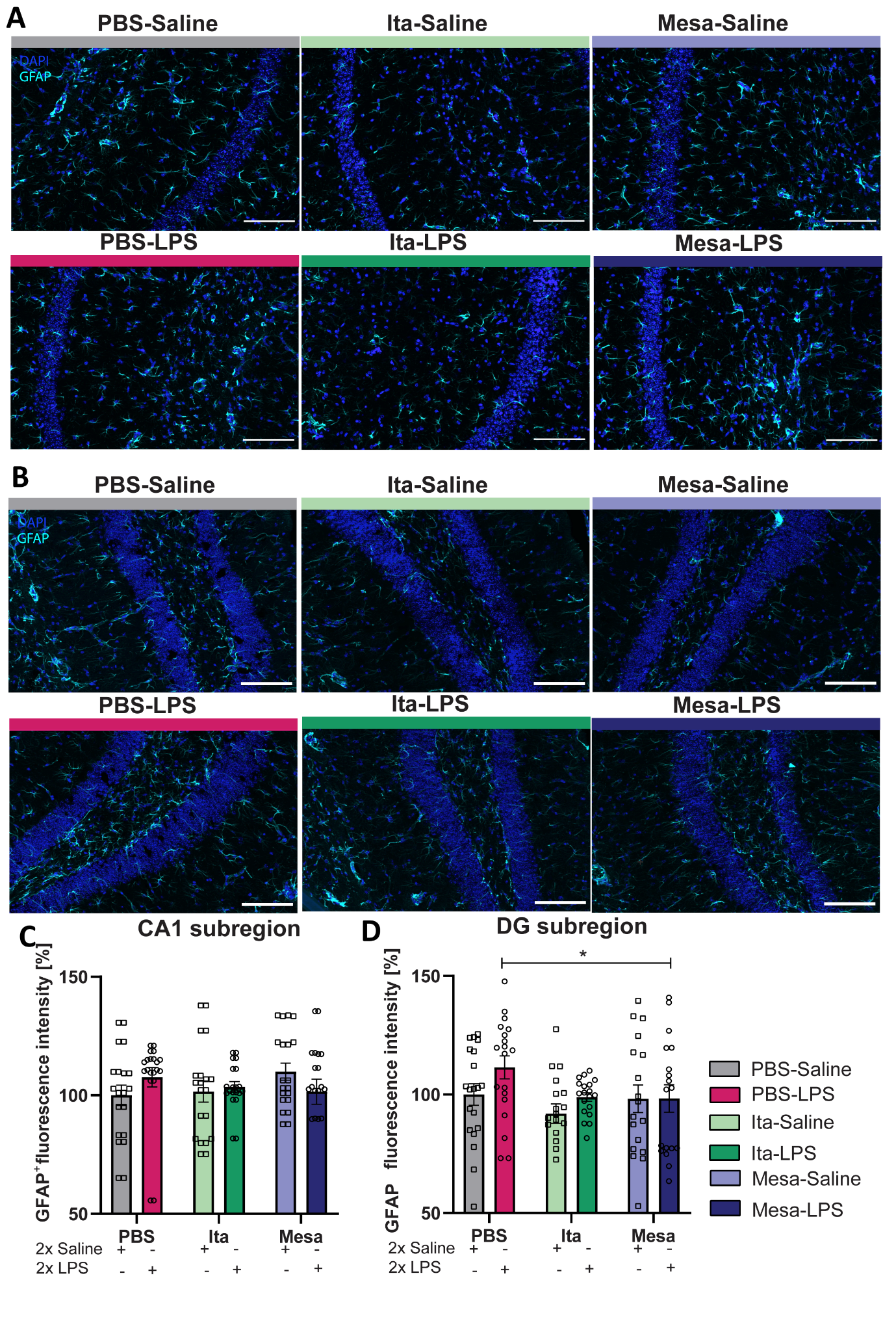


**Supplementary 1. Mesaconate slightly attenuate the LPS-induced increase in GFAP expression in astrocytes in the DG.** Immunohistochemical staining for GFAP and DAPI in the CA1 (A) and DG (B) the hippocampus for each experimental group (magnification 20x, scale bar is 100µm). (C,D) GFAP fluorescence intensity in the CA1 (C) and DG (D) of the hippocampus. Data are presented as mean±SEM and were analyzed with an ordinary two-way ANOVA followed by Fisher's LSD test; *p<0.05, [N (number of mice per group) = 4, CA1 n (number of analyzed images per group) = 20; DG n = 18-20].


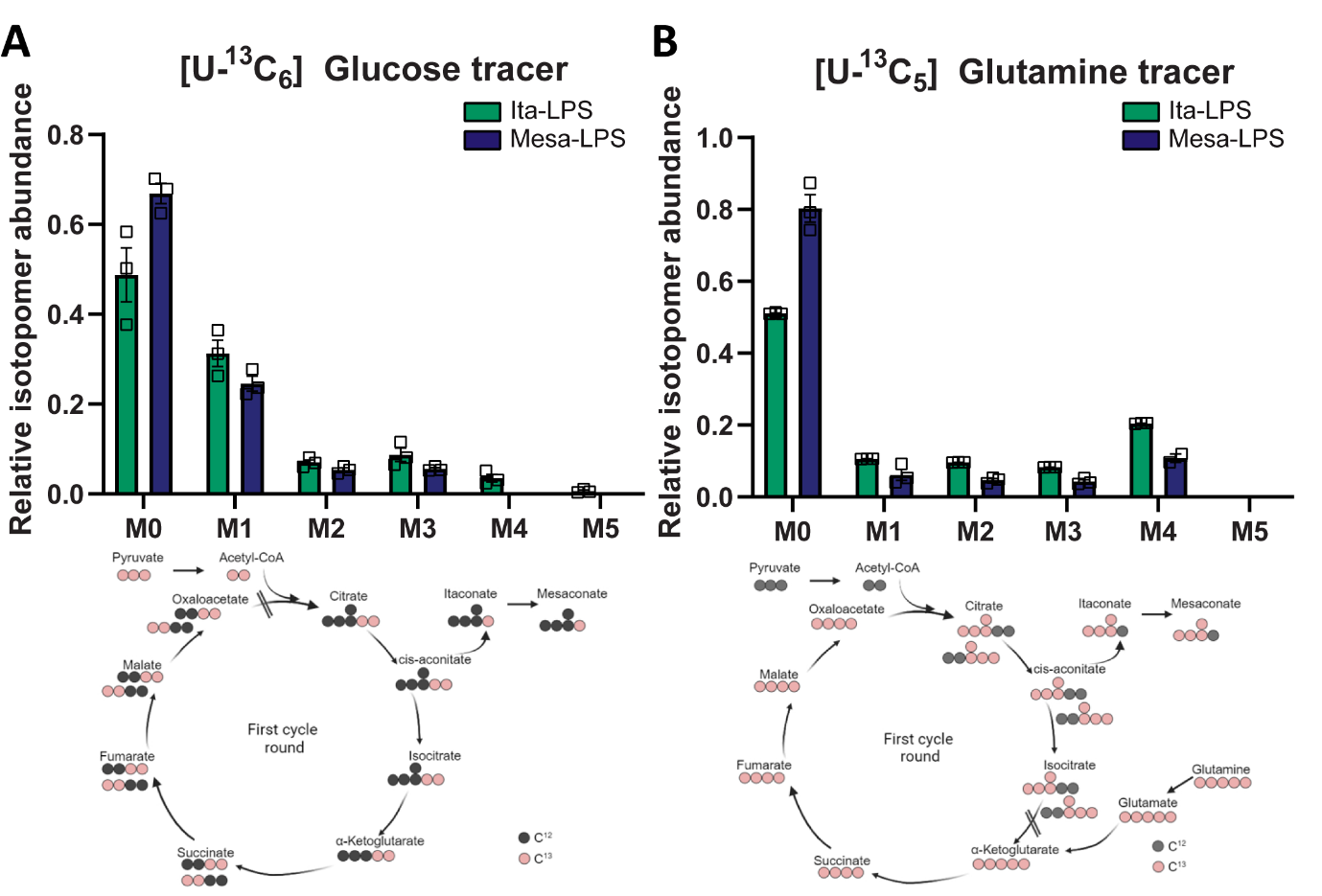


**Supplementary 2. Mesaconate is synthesized from itaconate in microglia** (A) Mass isotopomer distribution of itaconate and mesaconate in LPS stimulated primary microglia cells incubated with [U-13C] glucose tracer (right panel) or with [U-13C5] glutamine tracer (left panel) for twenty-four hours. Lower panel: Illustration of stable-isotope glucose labelling pattern in the TCA cycle with assumed reaction from itaconate to mesaconate. Data are presented as (A) mean±SEM of three independent experiments and (B) one experiment with three biological replicates.


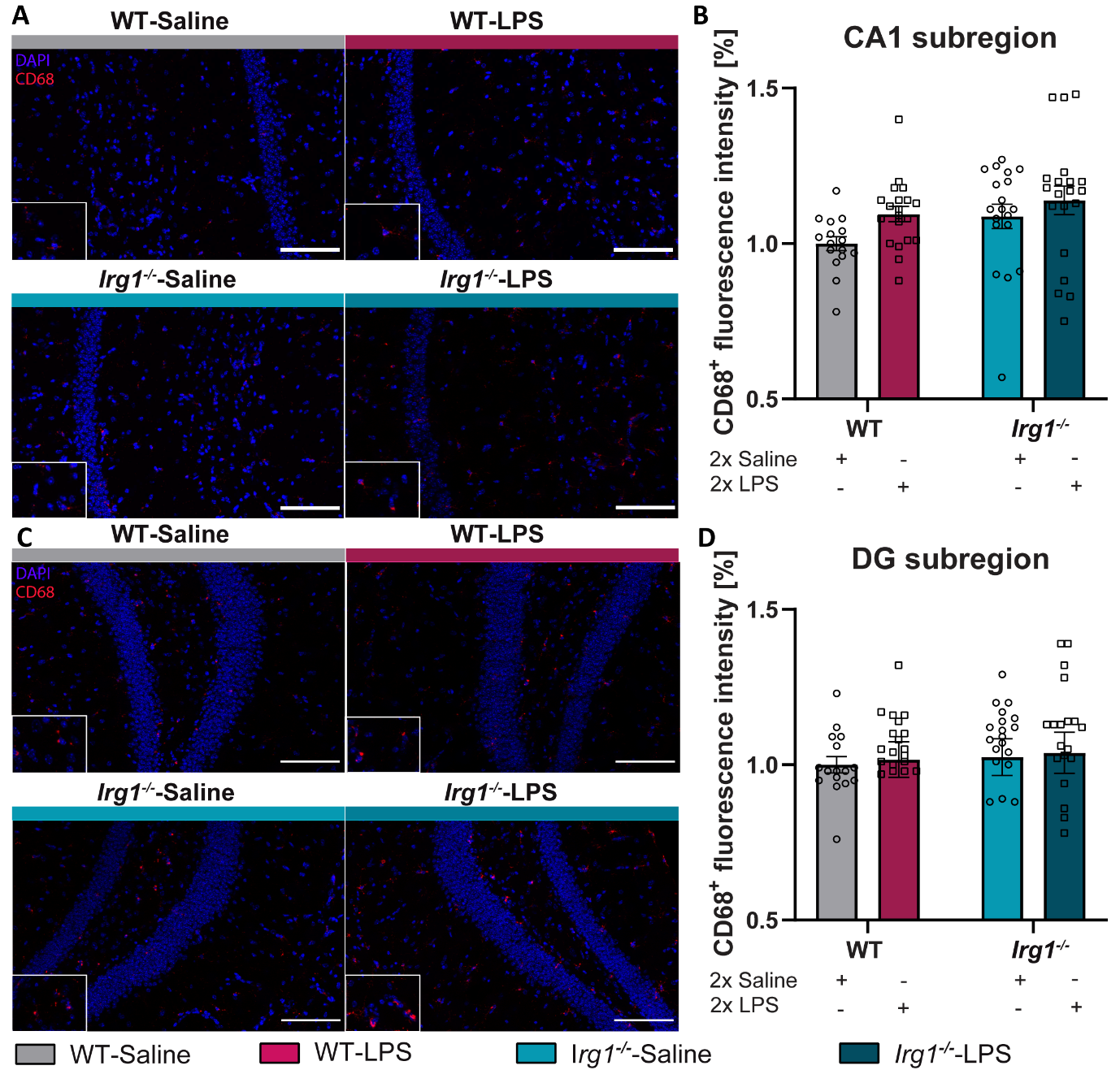


**Supplementary 3. LPS moderately increases CD68 in WT and *Irg1^-/-^* mice in the hippocampal CA1 region.** Representative images present immunohistochemical staining for CD68 and DAPI in the hippocampal CA1 (A) and DG (C) across all experimental groups (magnification 20x, scale bar is 100 µm). CD68 fluorescence intensity in the CA1 (B) and DG (D). Data are presented as mean±SEM and were analyzed with the repeated measures two-way ANOVA followed by Fisher's LSD test; [B-C: N (number of mice per group)=4-5 per group, CA1 n (number of analyzed imaged per group) =16–20, DG n=16–20].


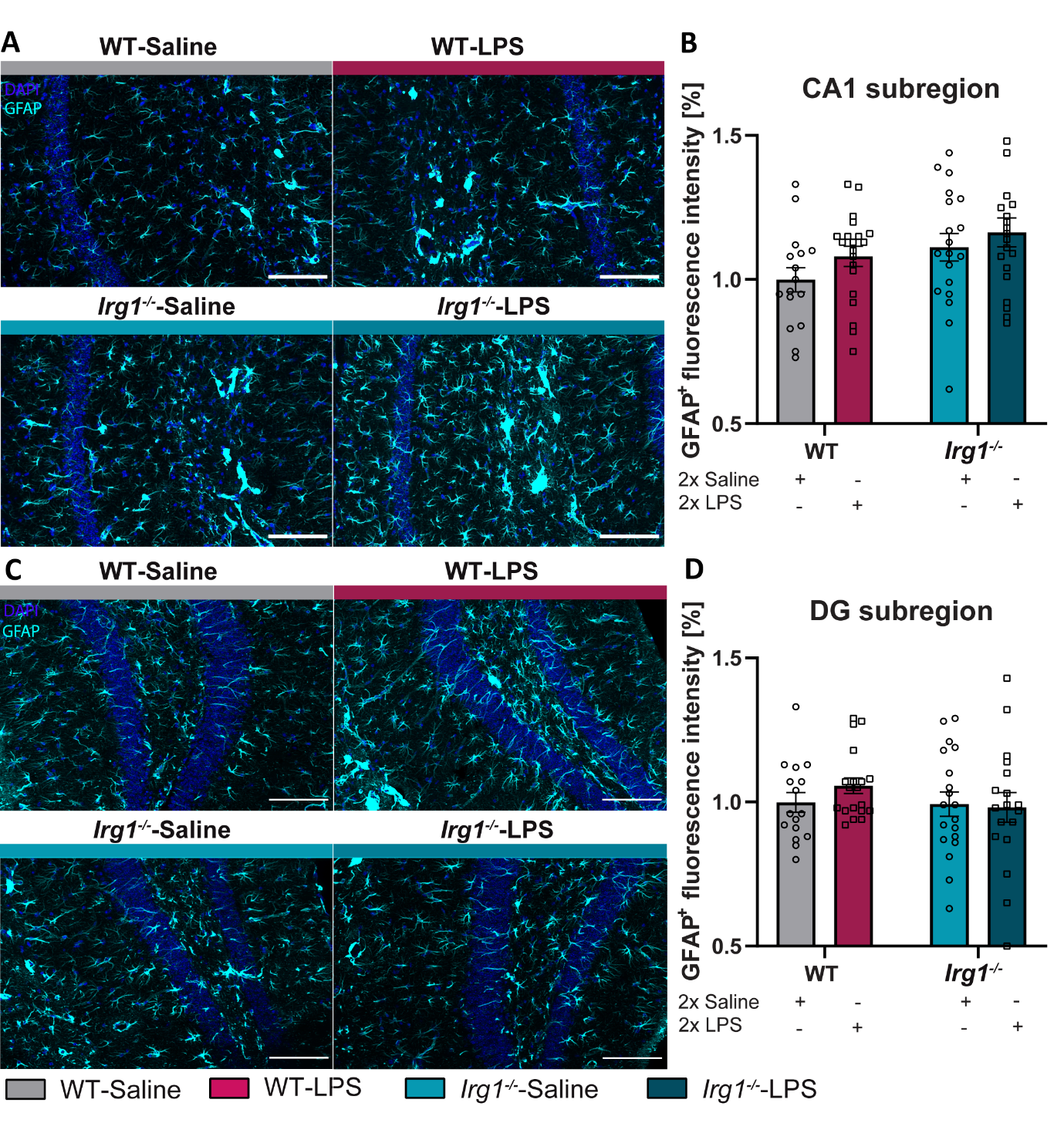


**Supplementary 4. LPS do not significantly increase GFAP^+^ intensity in WT and *Irg1^-/-^* mice. (A)** Representative images present immunohistochemical staining for GFAP and DAPI in the hippocampal CA1 (A) and DG (C) across all experimental groups (magnification 20x, scale bar is 100 µm). GFAP fluorescence intensity in the CA1 (B) and DG (D). Data are presented as mean±SEM and were analyzed with the repeated measures two-way ANOVA followed by Fisher's LSD test; [B,D: N (number of mice per group)=4-5 per group, CA1 n (number of analyzed imaged per group) =16–20, DG n=16–19].
